# APOE4/4 is linked to damaging lipid droplets in Alzheimer’s disease microglia

**DOI:** 10.1038/s41586-024-07185-7

**Published:** 2024-03-13

**Authors:** Michael S. Haney, Róbert Pálovics, Christy Nicole Munson, Chris Long, Patrik K. Johansson, Oscar Yip, Wentao Dong, Eshaan Rawat, Elizabeth West, Johannes C. M. Schlachetzki, Andy Tsai, Ian Hunter Guldner, Bhawika S. Lamichhane, Amanda Smith, Nicholas Schaum, Kruti Calcuttawala, Andrew Shin, Yung-Hua Wang, Chengzhong Wang, Nicole Koutsodendris, Geidy E. Serrano, Thomas G. Beach, Eric M. Reiman, Christopher K. Glass, Monther Abu-Remaileh, Annika Enejder, Yadong Huang, Tony Wyss-Coray

**Affiliations:** 1 Department of Neurology and Neurological Sciences, Stanford University School of Medicine, Stanford, CA USA; 2 Wu Tsai Neurosciences Institute, Stanford University, Stanford, CA USA; 3 Department of Materials Science & Engineering Department, Stanford University School of Medicine, Stanford, CA USA; 4 Gladstone Institute of Neurological Disease, Gladstone Institutes, San Francisco, CA USA; 5 The Institute for Chemistry, Engineering & Medicine for Human Health (ChEM-H), Stanford University, Stanford, CA USA; 6 Department of Cellular and Molecular Medicine, University of California, San Diego, San Diego, CA USA; 7 Development and Stem Cell Biology Graduate Program, University of California, San Francisco, CA USA; 8 Laboratory of Neuropathology, Banner Sun Health Research Institute, Sun City, AZ USA; 9 Banner Alzheimer’s Institute and Arizona Alzheimer’s Consortium, Phoenix, AZ USA; 10 Biomedical Sciences Graduate Program, University of California, San Francisco, CA USA; 11 Department of Neurology, University of California, San Francisco, CA USA; 12 The Phil and Penny Knight Initiative for Brain Resilience, Stanford University, Stanford, CA USA

**Keywords:** Alzheimer's disease, Microglia, Neuroimmunology

## Abstract

Several genetic risk factors for Alzheimer’s disease implicate genes involved in lipid metabolism and many of these lipid genes are highly expressed in glial cells^1^. However, the relationship between lipid metabolism in glia and Alzheimer’s disease pathology remains poorly understood. Through single-nucleus RNA sequencing of brain tissue in Alzheimer’s disease, we have identified a microglial state defined by the expression of the lipid droplet-associated enzyme ACSL1 with ACSL1-positive microglia being most abundant in patients with Alzheimer’s disease having the *APOE4/4* genotype. In human induced pluripotent stem cell-derived microglia, fibrillar Aβ induces *ACSL1* expression, triglyceride synthesis and lipid droplet accumulation in an APOE-dependent manner. Additionally, conditioned media from lipid droplet-containing microglia lead to Tau phosphorylation and neurotoxicity in an APOE-dependent manner. Our findings suggest a link between genetic risk factors for Alzheimer’s disease with microglial lipid droplet accumulation and neurotoxic microglia-derived factors, potentially providing therapeutic strategies for Alzheimer’s disease.

## Abstract (web summary)

A microglial state, featuring lipid droplets and secretion of neurotoxic factors, is shown to be most prominent in people with Alzheimer’s disease who have the *APOE4* genotype.

## Main

Alois Alzheimer’s original description of what would later be known as Alzheimer’s disease (AD) included the identification of “many glial cells show[ing] adipose saccules” in the brains of patients with dementia^2^. The description of this glial–lipid pathological hallmark of AD was made alongside the descriptions of the plaque and tangle pathology commonly associated with AD, yet the glial–lipid hallmark of the disease has received relatively little attention in AD research. A recent meta-analysis of all genetic risk factors for AD identified through genome-wide association studies discovered genes involved in lipid processing and innate immunity as a statistically enriched category of genetic risk factors for AD, alongside the more characteristic categories of genes in amyloid and Tau processing^1^. However, the role lipids and innate immunity play in AD risk remains poorly understood. *APOE* is one such lipid-related AD risk gene, which is highly upregulated in human microglia in AD^3^, and human induced pluripotent stem (iPS) cell-derived microglia (iMG) with *APOE* risk variants have more lipid droplets (LDs)^4^. Aged mouse microglia accumulate LDs and exhibit a dysfunctional microglial state termed LD-accumulating microglia (LDAM)^5^ and LDAM were also observed in chimaeric human–mouse AD models^6^. LDs form in myeloid cells through the upregulation of lipid-synthesis enzymes triggered by the engagement of toll receptors by innate immune triggers, such as bacteria^7^. LDs themselves have antimicrobial properties and are an evolutionarily conserved form of innate immune defence in macrophages^8^. Cholesterol-rich lysosomes and LDs in dysfunctional microglia have also been observed in the context of demyelination mouse models and human iPS cell models^9–12^. It remains unclear whether the lipid-accumulating glial state originally described by A. Alzheimer in human AD brain tissue is influenced by lipid AD risk variants (for example, *APOE*), if lipid-accumulating glia reported in AD are similar to recently identified LDAM and if lipid-accumulating glia play a benign, protective or damaging role in AD pathogenesis.

## Lipid-associated *ACSL1*^+^ microglia in AD

**Fig. 1:**
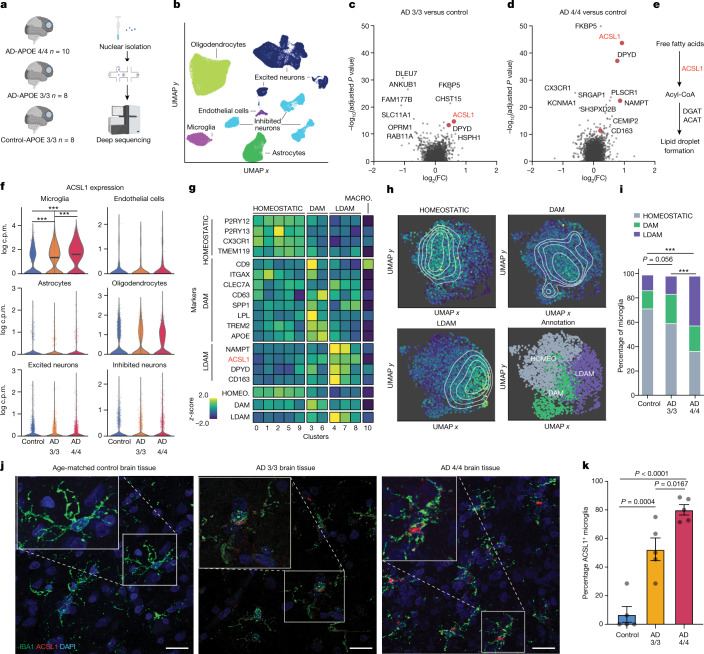
AD microglia have lipid transcriptional state defined by ACSL1. **a**, Schematic of snRNA-seq cohort and workflow (Methods). **b**, UMAP representation of all cells (*n* = 100,317) from snRNA-seq, coloured by annotated cell type. Data are shown after quality control and batch correction. **c**,**d**, Volcano plot representing MAST-based single-cell differential gene expression results (see section on ‘Single-cell differential gene expression’) of microglia from control individuals compared to microglia from individuals with AD and the *APOE3/3* genotype (**c**) and from individuals with AD and the *APOE4/4* genotype (**d**). Selected lipid- and metabolism-associated genes highlighted in red. **e**, Pathway diagram showing placement of differentially expressed gene *ACSL1* in pathway starting from free fatty acid to LD formation. **f**, Violin plots showing *ACSL1* expression across the cell types in the snRNA-seq dataset. Significance results indicate MAST-based adjusted *P* values (see section on ‘Single-cell differential gene expression’). **g**, Normalized and *z*-scored gene expression amounts of HOMEOSTATIC, DAM, LDAM and MACRO (macrophage) marker genes across the 11 subclusters identified in the microglia. HOMEOSTATIC, DAM and LDAM signature scores are shown across the 11 identified subclusters at the bottom. **h**, UMAP representation of microglia cells indicating the marker gene-based cell state annotation (bottom right) and the signature scores per cell for HOMEOSTATIC (top left), DAM (top right) and LDAM (bottom left) states. Contour lines indicate kernel density estimates of the signatures across the UMAP space. **i**, Bar plots indicating the percentage of cells from the three different cellular states (HOMEOSTATIC, DAM and LDAM) across microglia from control, AD-APOE3/3 and AD-APOE4/4 groups. Chi-square test results indicate the significance of the percentage differences between the groups (***P < 0.0001). **j**, Representative immunofluorescence images of human frontal cortex adjacent to the tissue used in snRNA-seq experiments stained for microglia marker IBA1 (green), *ACSL1* (red) and DAPI (blue) in an aged-matched healthy control subject (left), an AD-APOE3/3 subject (middle) and an AD-APOE4/4 subject. Scale bars, 20 μm. **k**, Quantification of percentage of IBA1^+^ microglia positive for *ACSL1*. *n* = 5 per group; each dot represents individual subject; one-way analysis of variance (ANOVA); mean ± s.e.m. Schematics in **a** created with BioRender.com.

**Extended Data Fig. 1:**
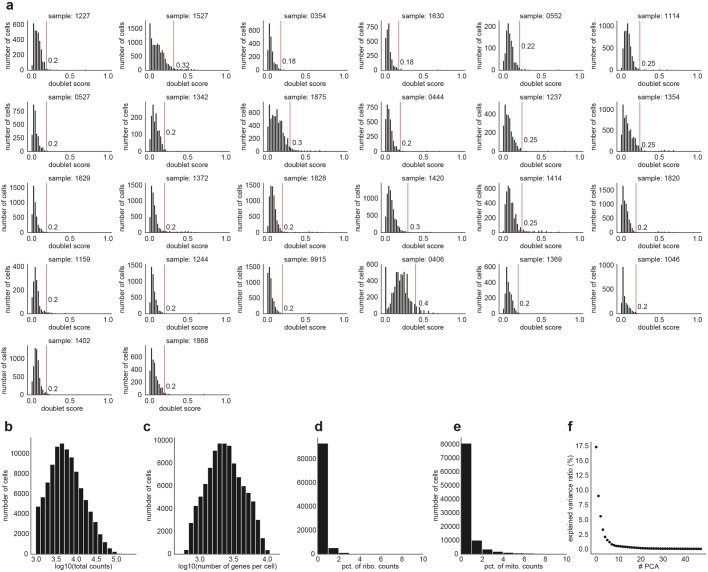
Quality Control for single-nucleus RNA-sequencing data. **a**, Doublet score distributions obtained with the Scrublet (0.2.3) Python package. Vertical lines indicate the doublet score thresholds identified per sample based on the distributions shown. **b**-**e**, Distribution of total read counts (**b**), number of genes expressed per cell (**c**), percent of reads mapped to ribosomal genes (**d**) and percent of reads mapped to mitochondrial genes (**e**) within the processed data after quality control. **f**, Percent of explained variance across the first 48 principal components.

**Extended Data Fig. 2:**
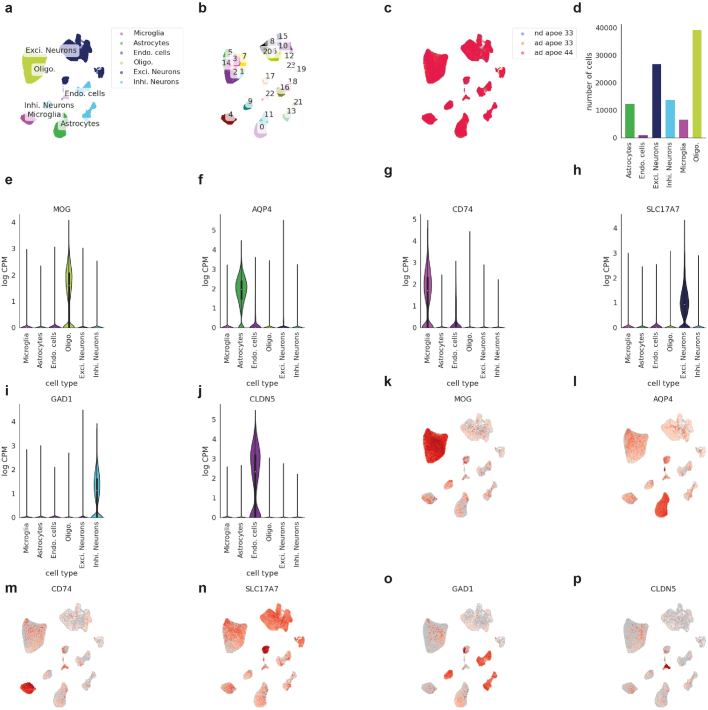
Annotations of CNS cell types from single-nucleus RNA-sequencing data. **a**-**c**, UMAP visualization of the whole snRNA-seq dataset after quality control and batch correction (n = 100,317). Cells are coloured by cell type annotation (**a**), subclusters drawn for cell type annotation (**b**), subject groups (control, AD-APOE3/3, AD-APOE4/4) (**c**). **d**, Bar chart indicating the total number of cells per cell type. **e**-**j**, Violin plots indicating gene expression levels of marker genes used for cell type annotations across the 6 identified cell types (n = 100,317). Violin plots are centred around the median, with their shape representing cell distribution. **k**-**p**, UMAP visualization of the whole snRNA-seq dataset coloured by cell type marker gene expression levels per cell.

**Extended Data Fig. 3:**
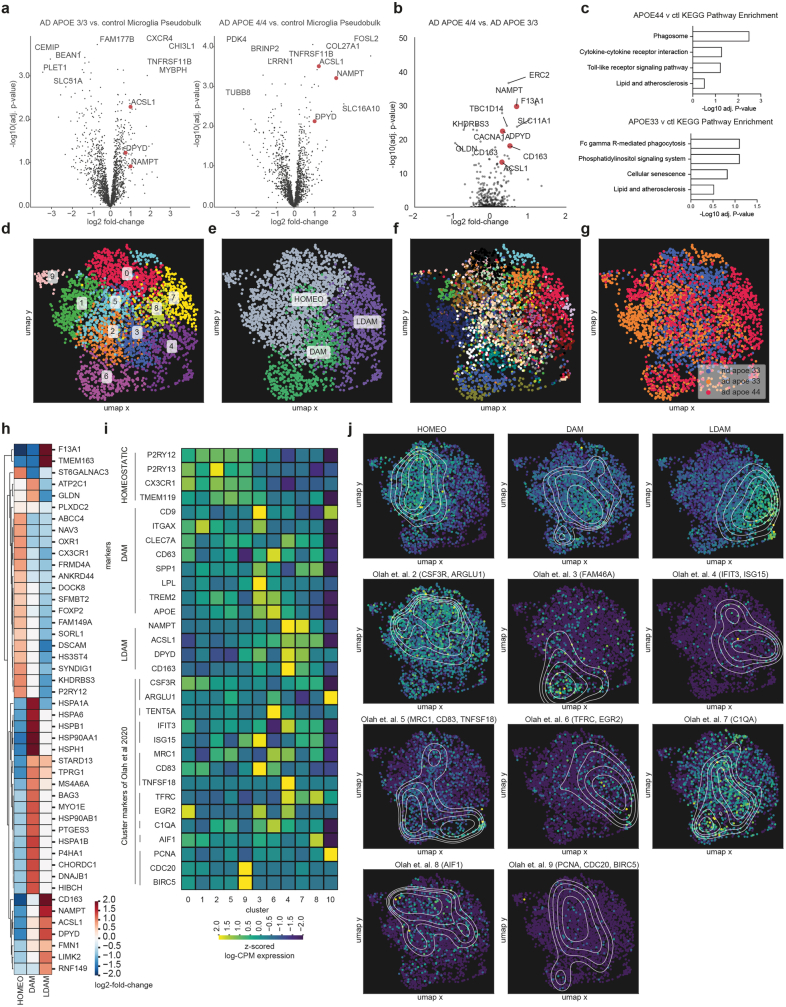
Differential expression analysis and subclustering of microglia from single-nucleus RNA-sequencing data. **a**, Volcano plot representing pseudobulk differential gene expression results (see Methods, Microglia pseudobulk differential gene expression) of microglia from control individuals compared to microglia (**left**) from subjects with AD and the *APOE3/3* genotype, (**right**) from subjects with AD and the *APOE4/4* genotype. Select lipid and metabolism-associated genes highlighted in red. **b**, Volcano plot representing single-cell differential gene expression results of microglia from subjects with AD and the *APOE3/3* genotype compared to microglia from subjects with AD and the *APOE4/4* genotype. Select lipid and metabolism-associated genes highlighted in red. **c**, Selected KEGG^61^ pathway analysis terms and enrichment score for top 200 differential expressed genes in between control and AD-APOE4/4 microglia (top) and control and AD-APOE3/3 microglia (bottom). **d**-**g**, UMAP visualization of the microglia coloured by identified subclusters (macrophage cluster 10 is not shown) (**d**), identified microglial states (**e**), subject IDs (**f**) and subject groups (control, AD-APOE3/3, AD-APOE4/4). **h**, Top marker genes identified for the 3 microglial states (HOMEOSTATIC, DAM, LDAM) with ‘one versus rest’ marker identification. Single-cell differential gene expression was performed with MAST (see Methods, Single-cell differential expression). Heatmap indicates significant (adj. p-value < 0.05) log2-fold changes. **i**, Normalized and z-scored gene expression levels of HOMEOSTATIC, DAM, LDAM marker genes as well as marker genes identified by Olah et al.^52^ across the 11 subclusters identified within the microglia. **j**, UMAP representation of microglia cells indicating signature scores per cell for HOMEOSTATIC, DAM, LDAM marker genes as well as marker genes identified by Olah et al.^52^ Contour lines indicate kernel density estimates of the signatures across the UMAP space.

To investigate the transcriptional state of postmortem human AD brain tissue in relationship to the APOE genotype we performed single-nucleus RNA sequencing (snRNA-seq) on fresh-frozen frontal cortex tissue from individuals diagnosed with AD with the *APOE4/4* genotype, individuals with AD and an *APOE3/3* genotype and age and sex-matched control individuals with the *APOE3/3* genotype (Fig. 1a and Supplementary Table 1). This yielded ~100,000 single-nucleus transcriptomes with all main cell types of the brain represented (Fig. 1b and Extended Data Figs. 1a–f and  2a–p). Differential gene expression analysis between control and AD-*APOE4/4* microglia revealed that the most significantly differentially expressed gene is acyl-CoA synthetase long-chain family member 1 (*ACSL1*), which encodes a lipid-processing enzyme (Fig. 1c,d, Extended Data Fig. 3a and Supplementary Table 2). *ACSL1* is a key enzyme in LD biogenesis and overexpression of *ACSL1* is sufficient to induce triglyceride-specific LD formation in several cell types^13,14^ (Fig. 1e). *ACSL1* was upregulated specifically in microglia in AD brain tissue compared to controls and to a greater extent in *APOE4/4* compared to AD-*APOE3/3* microglia (Fig. 1f, Extended Data Fig. 3b and Supplementary Table 2). Subclustering all the microglia from this study revealed that *ACSL1*^*+*^ microglia constitute a distinct state from homeostatic and disease-associated microglia (DAM) microglia, defined by the co-expression of more metabolic state regulators such as *NAMPT* and *DPYD* (Fig. 1g,h and Extended Data Fig. 3c–j). Owing to the set of LD-related genes in the *ACSL1*^+^ microglia cluster we refer to these *ACSL1*^+^ cells as LDAM. AD-*APOE4/4* brain tissue has the greatest percentage of LDAM, followed by AD-*APOE3/3* and the least amount of the LDAM microglia state is found in the aged-matched control brain tissue (Fig. 1i). Immunofluorescence microscopy of human AD brain tissue confirmed the *ACSL1* abundance differences observed by snRNA-seq (Fig. 1j,k).

## Lipid accumulation is linked to AD pathology

**Fig. 2:**
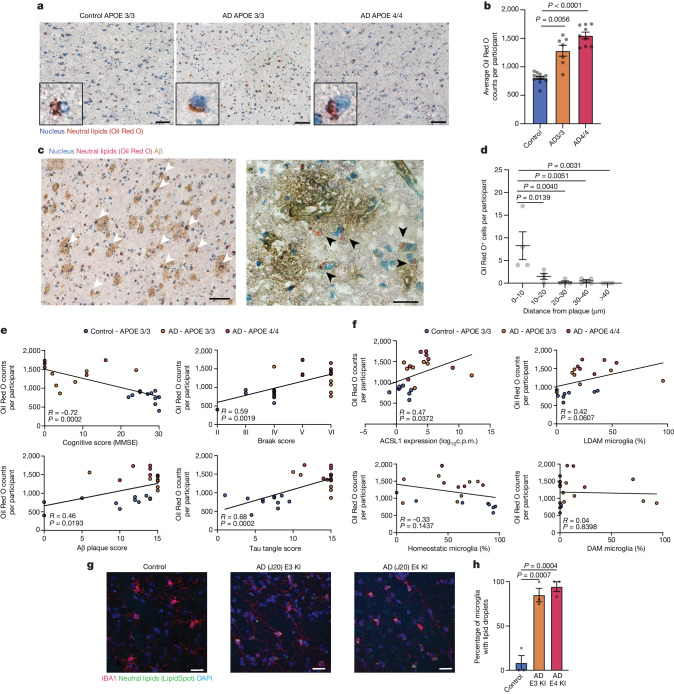
Lipid accumulation is linked to AD pathology. **a**, Representative Oil Red O staining image for control, AD-*APOE3/3* and AD-*APOE4/4* human frontal cortex. Neutral lipids stained with Oil Red O (red) and nuclei stained with haematoxylin (blue). Scale bars, 50 μm. **b**, Quantification of Oil Red O staining. Bar plots represent average Oil Red O counts per image for each individual category (control, *n* = 12; AD33 *n* = 7; AD44 *n* = 9 individuals). Each dot represents average Oil Red O counts for an individual averaged over five ×20 image fields per individual; one-way ANOVA; mean ± s.e.m. **c**, Left, Oil Red O staining of individuals with AD-*APOE4/4* and with IHC for Aβ. White arrowheads represent Oil Red O^+^ cells in or around Aβ plaques. Scale bar, 50 μm. Right, high magnification of representative Oil Red O stain with IHC for Aβ in an individual with AD-*APOE4/4*. Black arrowheads represent Oil Red O^+^ cells in or around Aβ plaques. Scale bar, 20 μm. **d**, Quantification of the frequency of Oil Red O^+^ cells in various distances from Aβ plaques (*n* = 4 per group; one-way ANOVA; mean ± s.e.m.). **e**, Scatter plot of average Oil Red O counts per individual averaged over five ×20 image fields per individual with individual’s metadata. Individual category coloured blue for control, orange for individuals with AD-*APOE3/3* and red for those with AD-*APOE4/4*. *P* values determined by Spearman correlation. **f**, Scatter plot of average Oil Red O counts per individual averaged over five ×20 image fields per individual with individual’s snRNA-seq data. Individual category coloured blue for control, orange for individuals with AD-*APOE3/3* and red for those with AD-*APOE4/4*. *P* values determined by Spearman correlation. **g**, Representative immunofluorescence images of mouse hippocampus tissue stained for microglia marker IBA1 (red), neutral lipids (LipidSpot, green) and DAPI (blue) in control age-matched non-transgenic mice (left), AD mouse model (J20) with human *APOE3* knockin (middle) and AD mouse model (J20) with human *APOE4* knockin (right). Scale bars, 20 μm. **h**, Quantification of average percentage of IBA1+ microglia with neutral lipid dye (LipidSpot) (*n* = 3 individual mice per group; one-way ANOVA; mean ± s.e.m).

**Extended Data Fig. 4:**
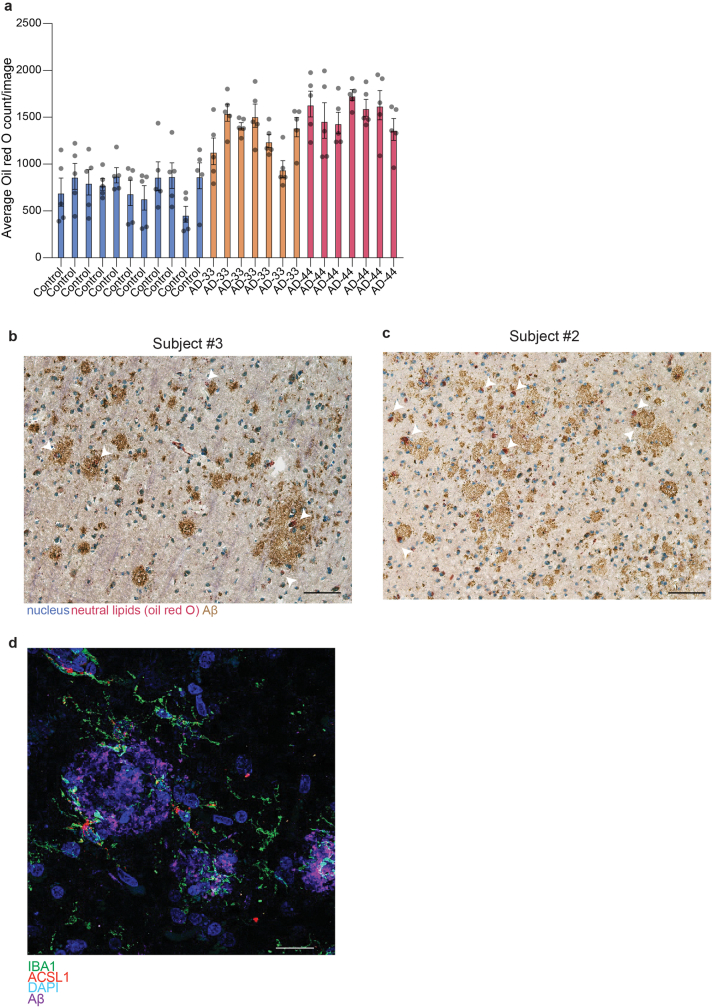
Additional microscopy and quantification of human AD brain tissue. **a**, Quantification of Oil Red O counts for each subject (n = 5 20x images per subject; mean ± s.e.m.). **b**-**c**, Representative Oil Red O staining of *APOE4/4* AD subjects with IHC for Aβ. White arrowheads represent Oil Red O positive cells in or around Aβ plaques. Scale bars (black, bottom right) 50 μm. **d**, Representative immunofluorescence images of human frontal cortex adjacent to tissue used in snRNA-seq experiments stained for microglia marker IBA1 (green), ACSL1 (red) and DAPI (blue) and amyloid-beta (magenta) in an AD *APOE4/4* subject. Scale bars (white, bottom right) 20 μm. Data in **b**-**d** replicated in at least two independent experiments.

To measure intracellular lipid accumulation, AD and control brain sections were stained with Oil Red O, a dye for neutral lipids. The brains of patients with AD-*APOE4/4* showed an abundance of perinuclear Oil Red O^+^ lipid bodies which resemble LD and are similar to Alzheimer’s original description of adipose saccules in glial cells of postmortem brain tissue of patients (Fig. 2a). These lipid bodies are most prevalent in AD brain tissue with a slight but not significant increase in individuals with AD-*APOE4/4* compared to those with AD-*APOE3/3* (Fig. 2b and Extended Data Fig. 4a). Oil Red O^+^ cells are often found near to or at the core of amyloid-β (Aβ) plaques (Fig. 2c,d and Extended Data Fig. 4b,c). In a similar fashion, ACSL1^+^ microglia are often observed near Aβ plaques (Extended Data Fig. 4d), suggesting that the cells containing lipid bodies near plaques could be ACSL1^+^ microglia. However, the identification of the cell type(s) which accumulate these lipids necessitates simultaneous staining for lipids and several protein markers, which is challenging in aged human histological brain sections with current technology.

The number of lipid bodies is negatively correlated with cognitive performance, as measured by the mini-mental state exam (MMSE) and positively correlated with Aβ plaque amounts and Tau pathology levels (Fig. 2e). Because Oil Red O staining and snRNA-seq were done on the same samples we were able to correlate gene expression with the abundance of lipid bodies for each sample. Reassuringly, the expression of *ACSL1* by microglia positively correlated with the relative numbers of lipid bodies (Fig. 2f). Mirroring these observations in the human AD tissue, there are more LD^+^ microglia in the J20/*APOE3* and J20/*APOE4* models of AD^15^ compared to age-matched wild-type mice (Fig. 2g,h).

## ACSL1 and triglycerides increase after fAβ challenge

**Fig. 3:**
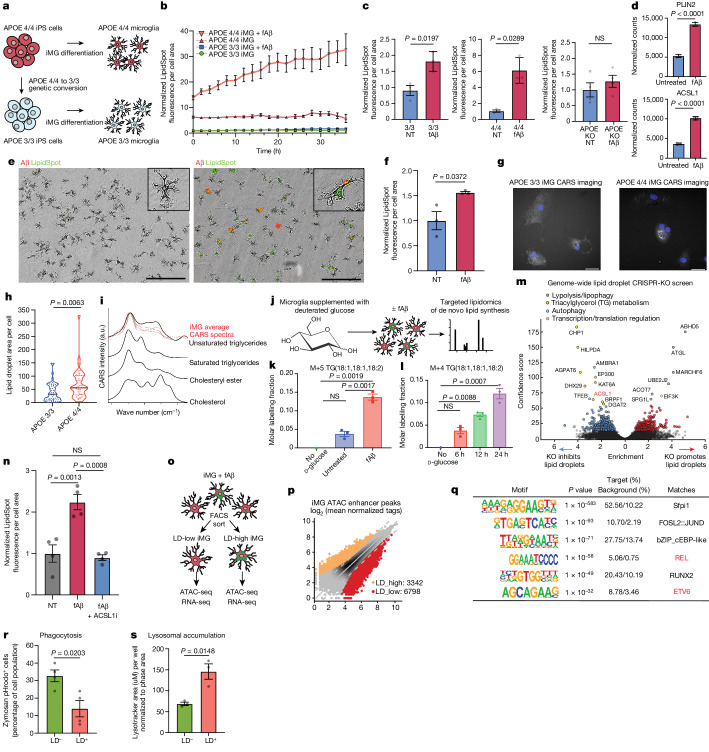
iMG increase ACSL1 and triglyceride lipid synthesis after fAβ challenge. **a**, Schematic of *APOE3/3* and *APOE4/4* iMGs. **b**, Quantification of lipid fluorescent dye (LipidSpot) in *APOE3/3* and *APOE4/4* iMG ± fAβ (*n* = 3 replicate wells per condition; mean ± s.e.m.). **c**, Average LipidSpot fluorescence per cell normalized to the not treated (NT) condition at final time point in **b**. Individual dots represent replicate wells (*n* = 3 replicate wells per condition; unpaired two-sided *t*-test; per condition, mean ± s.e.m). **d**, Normalized gene expression counts for significant differentially expressed genes in *APOE4/4* iMG ± fAβ (*n* = 3 replicate wells per condition, *P* values determined by DEseq2; mean ± s.e.m). **e**, Primary rat microglia untreated (left) or with fAβ (right) with LipidSpot. Scale bar, 200 μm. **f**, Average LipidSpot fluorescence per cell normalized to untreated images in **e** (*n* = 3 replicate wells per condition; unpaired two-sided *t*-test; mean ± s.e.m). **g**, CARS images of *APOE4/4* and *APOE3/3* iMG ± fAβ. Scale bars, 20 μm. Data replicated in at least two independent experiments. **h**, Quantification of CARS microscopy. Each dot represents lipid measurements from individual cells (APOE33 *n* = 47; APOE44 *n* = 38; unpaired two-sided *t*-test). **i**, CARS spectra from fAβ-treated iMG (red) and reference spectra for common lipid species (black). **j**, Schematic of lipidomics measurement of d-glucose^13^C incorporation in BV2 cells + fAβ. **k**, Incorporation of d-glucose^13^C into triglycerides in microglia ± fAβ (*n* = 3 replicate wells per condition; one-way ANOVA; mean ± s.e.m). **l**, Incorporation of d-glucose^13^C into triglycerides in Aβ-treated microglia over time (*n* = 3 replicate wells per condition; one-way ANOVA; mean ± s.e.m). **m**, Volcano plot of genome-wide CRISPR-KO LD screen in U937 cell line. Genes passing a 10% FDR cutoff are highlighted in red and blue. **n**, Average LipidSpot fluorescence ± ACSL1 inhibitor (Triacin C) (*n* = 4 replicate wells per condition; unpaired two-sided *t*-test; mean ± s.e.m.). **o**, Schematic of ATAC-seq and RNA-seq in LD-high and LD-low iMGs. **p**, ATAC-seq peaks in LD-high versus LD-low iMGs. **q**, Motif analysis of differential peaks. Motifs enriched in lipid-associated macrophages are highlighted in red. **r**, Average percentage pHrodo zymosan^+^ iMGs ± LD (*n* = 4 replicate wells per condition; unpaired, two-sided *t*-test; mean ± s.e.m.). **s**, Average percentage lysotracker^+^ iMGs ± LD (*n* = 3 replicate wells per condition; unpaired, two-sided *t*-test; mean ± s.e.m.). KO, knockout; NS, not significant. Elements in **j** created with BioRender.com.

**Extended Data Fig. 5:**
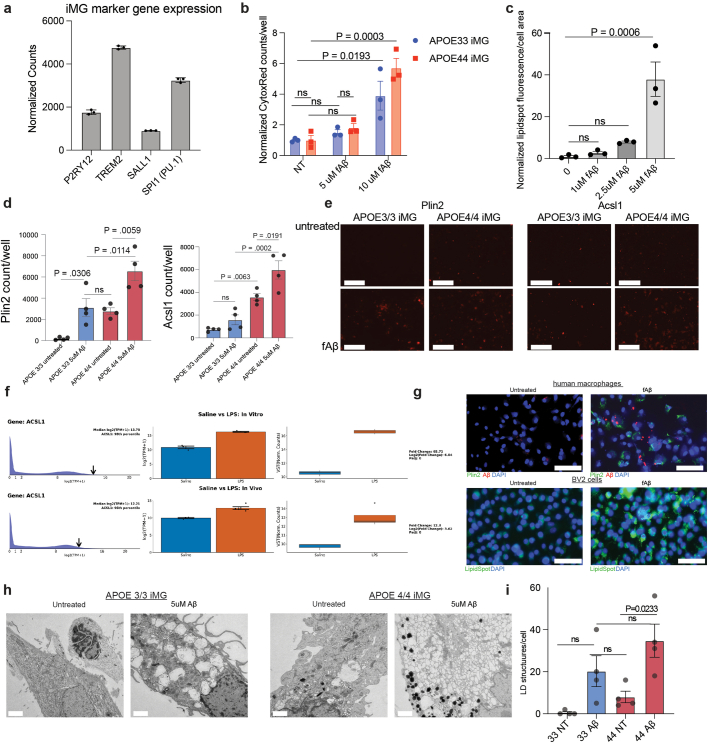
Additional in vitro experiments on lipid droplet production and ACSL1 expression after fAβ treatment. **a**, Expression of microglia marker genes in iMG (n = 3 replicate wells; mean ± s.e.m). **b**, Toxicity of fAβ at different concentrations in *APOE4/4* and *APOE3/3* iMG after 24 hours incubation followed by staining with CytoxRed. (n = 3 replicate wells per condition; one-way ANOVA; mean ± s.e.m.). **c**, Dose-dependent effect of fAβ on lipid accumulation (n = 3 replicate wells per condition; one-way ANOVA; mean ± s.e.m.). **d**-**e**, iMG treated with 5 μM Aβ for 24 hours (left) with *PLIN2* and *ACSL1* Immunofluorescence quantification with (**d**) with representative images (**e**) (n = 4 replicate wells per condition; one-way ANOVA; mean ± s.e.m.; scale bars (white, bottom left) 200 μm). **f**, *ACSL1* gene expression measured in human iMG after LPS treatment as described in Hasselmann et al. 2019^22^ (https://rnaseq.mind.uci.edu/blurton-jones/bulkSeq/); box plot centred at median with boarders representing quartiles. mean ± s.e.m. **g**, Representative image of human macrophages (top) untreated (left) or treated (right) with 5 μM Aβ for 24 hours (left) with *PLIN2* (green) and Aβ (red) staining. Scale bars (white, bottom right) 50 um. Representative image of mouse BV2 cells (bottom) untreated (left) or treated (right) with 5uM Aβ for 24 hours (left) with LipidSpot (green) and Aβ (red) staining. Scale bars (white, bottom right) 75 μm. Data in replicated in at least two independent experiments. **h**-**i**, Transmission electron microscopy of *APOE3/3* iMG and *APOE 4/4* iMG treated with 5 μM Aβ for 24 hours and untreated iMG (Scale bars (white, bottom right) 2 μm; n = 4 replicate wells per condition; one-way ANOVA; mean ± s.e.m.).

To directly test whether the *APOE* genotype contributes to LD accumulation in microglia, *APOE4/4* and isogenic *APOE3/3* iPS cells^16^ were differentiated into microglia (iMG) as previously described^17–19^ (Fig. 3a and Extended Data Fig. 5a). Live cell microscopy of iMG with a fluorescent dye for neutral lipids (LipidSpot) showed greater LD accumulation in *APOE4/4* iMG compared with isogenic *APOE3/3* iMG, similar to recent reports in isogenic iMG^4^ and astrocytes^20^. However, treatment of iMG with fibrillar Aβ (fAβ) led to a strong increase in LD accumulation which was exacerbated by the presence of the *APOE4* AD-risk allele. The effect of fAβ on LD accumulation was absent in the *APOE*-KO background (Fig. 3b,c and Extended Data Fig. 5b,c). In accordance with this accumulation of LDs, the gene expressions of LD-associated genes *PLIN2* (ref. ^21^) and *ACSL1* (refs. ^13,14^) are upregulated on fAβ challenge in iMGs (Fig. 3d) and to a greater extent in the *APOE4/4* background (Extended Data Fig. 5d,e). A previously published dataset shows that *ACSL1* is highly upregulated by the innate immune trigger lipopolysaccharides (LPS) in an independent human iMG chimaeric mouse model^22^ (Extended Data Fig. 5f), suggesting that *ACSL1* upregulation in microglia is the consequence of a broader response to innate immune triggers. To ensure the LD induction in microglia by fAβ is not unique to these iPS cell lines or the differentiation protocol, primary rat microglia were treated with fAβ and LD accumulation was also observed (Fig. 3e,f). The induction of LDs by fAβ was also observed in primary human macrophages and the mouse BV2 microglial cell line (Extended Data Fig. 5g). In addition to lipid dyes to measure LD accumulation, transmission electron microscopy indicated an increase in LD concentrations in iMG on fAβ challenge (Extended Data Fig. 5h,i). We performed coherent anti-Stokes Raman scattering (CARS) imaging on iMG to confirm differential lipid accumulation between the *APOE* genotypes after the fAβ challenge. Analysis of the CARS imaging of fAβ-treated iMGs revealed that the LD spectra overlap with unsaturated (triglyceride) spectra (Fig. 3g–i). To investigate if these lipids are synthesized de novo in response to fAβ, BV2 microglia were grown with deuterated glucose (d-glucose) (Fig. 3j). Lipidomic analysis showed a time-dependent increase in triglyceride incorporation of d-glucose after fAβ challenge (Fig. 3k,l). To assess which specific lipid synthesis genes in the human genome play a role in LD accumulation, we performed a genome-wide CRISPR-KO screen in the monocyte cell line U937 by FACS. This screen revealed regulators of triglyceride metabolism as being a top category of genes required for LD accumulation and *ACSL1* as one of the most significant genes required for LD formation (Fig. 3m and Supplementary Table 3). An *ACSL1* inhibitor (Triacin C) reversed the accumulation of LD in *APOE4/4* iMG on fAβ challenge (Fig. 3n).

**Extended Data Fig. 6:**
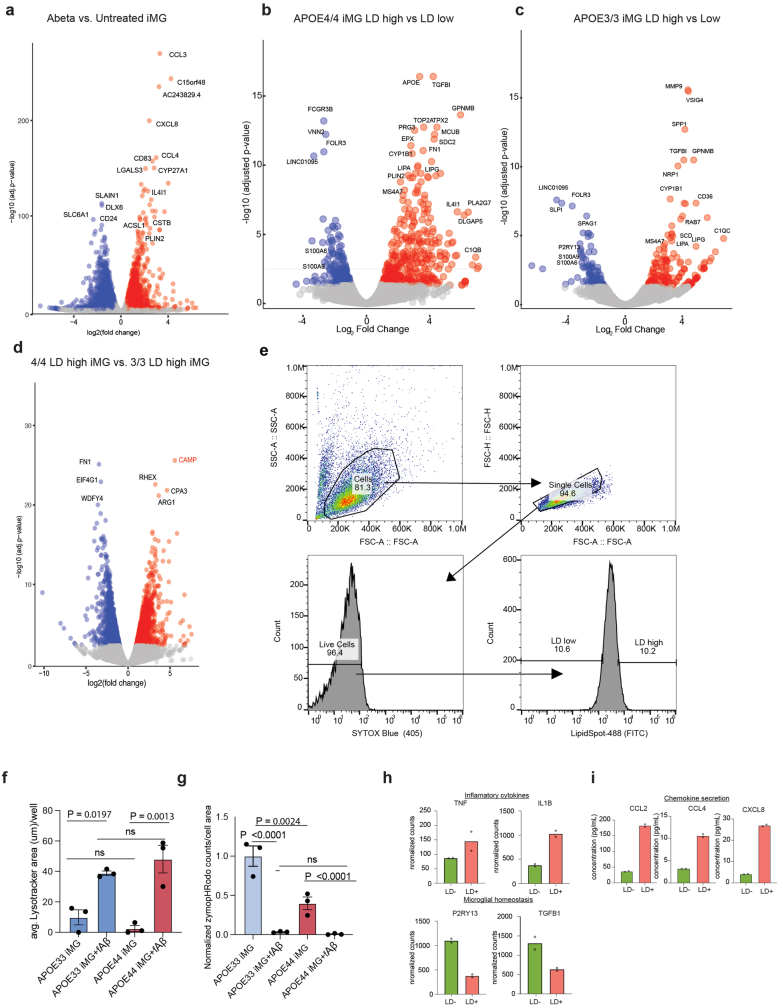
Bulk RNA-saq of iMG and phenotypic assays. **a**, Volcano plot of differential gene expression analysis of untreated and Aβ treated *APOE4/4* iMG. **b**, Volcano plot of differential gene expression analysis of lipid droplet high and lipid droplet low *APOE4/4* iMG. **c**, Volcano plot of differential gene expression analysis of lipid droplet high and lipid droplet low *APOE3/3* iMG. **d**, Volcano plot of differential gene expression analysis of lipid droplet high *APOE3/3* iMG and lipid droplet high *APOE4/4* iMG. **e**, Example gating scheme for separating cells based on lipid droplet content. **f**, LysoTracker area per cell after incubation with fAβ in *APOE3/3* and *APOE4/4* iMG (n = 3 replicate wells per condition; one-way ANOVA; mean ± s.e.m.). **g**, Phrodo zymosan phagocytosis area per cell after incubation with fAβ in *APOE3/3* and *APOE4/4* iMG (n = 3 replicate wells per condition; one-way ANOVA; mean ± s.e.m.). **h**, Normalized gene expression counts for significant DEGs in LD-high versus LD-low iMGs (n = 2 replicate wells per condition). **i**, Secreted chemokines in LD-high versus LD-low iMGs (n = 2 replicate wells per condition).

To assess the transcriptomic and epigenetic state of microglia with LD accumulation we performed FACS sorting of LD-high and LD-low iMG followed by ATAC-seq and RNA-seq (Fig. 3o,p, Extended Data Fig. 6a–e and Supplementary Tables 4 and 5). We detected 3,442 peaks which were gained in LD-high microglia compared to LD-low microglia. ATAC-seq peaks at enhancer regions for LD-high were highly enriched for microglia lineage-determining transcription factors for PU.1. In addition, peaks specific for LD-high microglia showed enrichment for motifs related to the NF-κB family of transcription factors (for example, REL and ETV6) (Fig. 3q). This epigenetic signature is also seen in macrophages in atherosclerosis models^23^ and adipose-associated macrophages^24^ but this signature is not seen in DAM microglia^25^. RNA-seq results indicated that LD-high microglia had higher expression of NF-κB associated pro-inflammatory cytokines (for example, *TNFA* and *IL1B*) and lower expression of microglial homeostasis markers compared to LD-low microglia (Extended Data Fig. 6a–c,h). Interestingly, among the top differentially expressed genes between *APOE3/3* LDAM and *APOE4/4* LDAM, we identified the antibacterial LD-associated protein cathelicidin or CAMP (Extended Data Fig. 6d and Supplementary Table 4), which is found on LD surfaces with antimicrobial properties in macrophages exposed to bacteria^8^. In accordance with these RNA-seq results, phenotypic measurements of LD-containing *APOE4/4* iMGs indicate that they are dysfunctional in phagocytosis, accumulate lysosomes and secrete inflammation-associated chemokines as measured in the cell culture media (Fig. 3r,s and Extended Data Fig. 6f,g,i). These results are similar to recent reports describing mouse LDAM that have a dysfunctional and inflammatory microglial state^5^.

**Extended Data Fig. 7:**
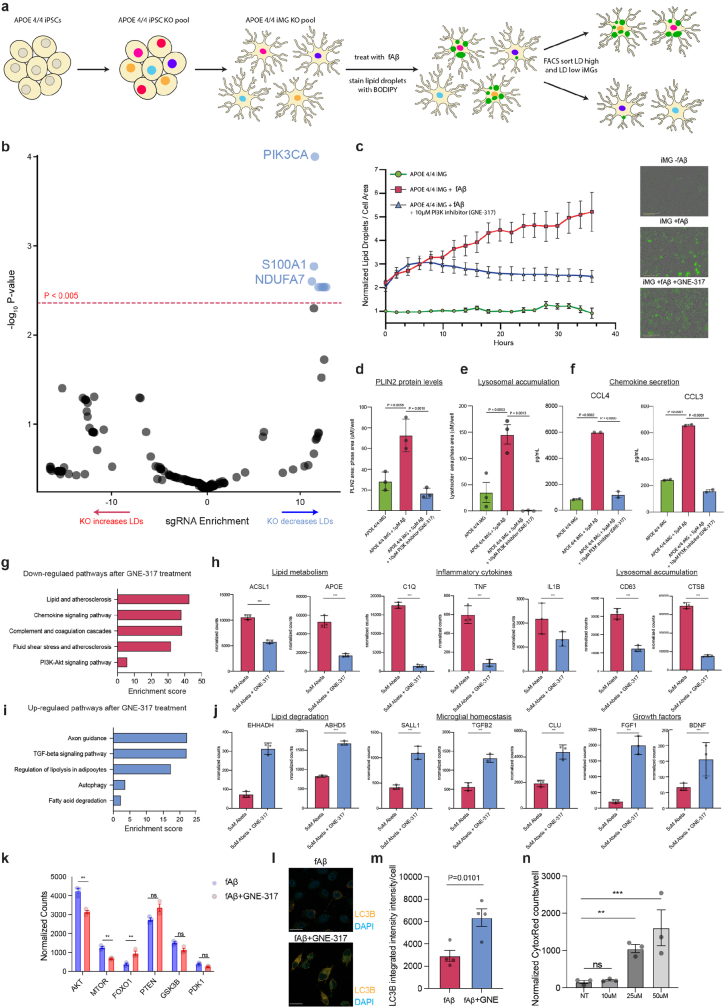
CRISPR-KO screen in iMG for LD levels and effect of PIK3CA inhibition on LD levels. **a**, Schematic of CRISPR-KO screen in *APOE4/4* iMG for lipid droplet formation following Aβ treatment. **b**, Volcano plot representing CRISPR screen results. Effect score represents log_2_ fold change in sgRNA counts in lipid droplet negative versus lipid droplet-positive cell fraction. Screen hits with P-value < 0.005 coloured blue. **c**, Live cell imaging of untreated *APOE4/4* iMG, 5 μM Aβ treated iMG and 5 μM Aβ treated iMG with 10 μM GNE-317. The y axis represents average green fluorescence per cell normalized to untreated *APOE4/4* iMG at the first time point and the x-axis represents imaging time points in hours (n = 3 replicate wells per condition; mean ± s.e.m.) (left). Representative images at the final time point (right) with LipidSpot signal represented in green. **d**, Quantification of *PLIN2* Immunofluorescence in untreated, Aβ treated and Aβ treated with 10 uM GNE-317 conditions iMG for 24 hours. (n = 3 replicate wells per condition; one-way ANOVA; mean ± s.e.m.). **e**, Quantification of lysotracker staining in untreated, Aβ treated and Aβ treated with 10 μM GNE-317 conditions iMG for 24 hours (n = 3 replicate wells per condition; one-way ANOVA; mean ± s.e.m.). **f**, Measurement of secreted chemokines in cell culture media in untreated, Aβ treated and Aβ treated with 10 μM GNE-317 conditions iMG for 24 hours. Individual dots represent replicate wells (n = 2, one-way ANOVA; mean ± s.e.m.). **g**, Selected KEGG pathway enrichment terms for the top 200 significant downregulated genes ranked by p-value upon GNE-317 treatment in *APOE4/4* iMG when challenged with Aβ. **h**, Normalized gene expression counts for significantly downregulated genes with GNE-317 treatment in *APOE4/4* iMG when challenged with Aβ *APOE4/4* iMG (n = 3 replicate wells per condition, mean ± s.e.m., *** P < 0.0001). P values determined by DEseq2. **i**, Selected KEGG pathway enrichment terms for top 200 significant upregulated genes ranked by p-value upon GNE-317 treatment in *APOE4/4* iMG when challenged with Aβ. **j**, Normalized gene expression counts for significantly upregulated genes with GNE-317 treatment in *APOE4/4* iMG when challenged with Aβ *APOE4/4* iMG (n = 3 replicate wells per condition; mean ± s.e.m.; *** P < 0.0001). P values determined by DEseq2. **k**, Differential gene expression for genes in mTOR and autophagy pathways upon GNE-317 treatment with fAβ challenge in iMG. (n = 3 replicate wells per condition; mean ± s.e.m.;*** P < 0.0001). P values determined by DEseq2. **l**, Representative images of LC3B Immunofluorescence (yellow) upon fAβ and GNE-317 treatment. The scale bar (white, bottom left) represents 20μm. **m**, Quantification of LC3B immunofluorescence by integrated fluorescence intensity per DAPI signal. Individual dots represent replicate wells (n = 4 per condition; two-sided, t-test; mean ± s.e.m.). **n**, Toxicity measurements of GNE-317 as determined by cytox-red. (n = 4 per condition; mean ± s.e.m; one-way ANOVA).

To discover genetic modifiers of LD accumulation in iMG by fAβ, we performed a CRISPR-KO screen in *APOE4/4* iMG with a library of about 20,000 single guide RNAs (sgRNAs) targeting the ‘druggable genome’ of about 2,000 genes representing all human kinases, phosphatases and known drug targets (Extended Data Fig. 7a). The top hit from this screen was *PIK3CA*, a catalytic subunit of PI3 kinase (Extended Data Fig. 7b and Supplementary Table 6). Interestingly, the second top hit was S100A1, an AD risk gene downstream of the LPS and TLR4 response in macrophages. PI3 kinase inhibition is a known modulator of LDs in mouse macrophages^26^ but this has not been previously shown in human microglia. We tested if inhibition of PI3 kinase would reduce LD accumulation in iMGs. Indeed, the small-molecule PI3K inhibitor GNE-317 dramatically reduced lipid droplet formation in *APOE4/4* iMGs exposed to fAβ quantified by live microscopy and *PLIN2* immunofluorescence (Extended Data Fig. 7c,d). In addition, PI3K inhibition with GNE-317 reversed the lysosomal accumulation and inflammatory cytokine secretion observed in iMGs with high concentrations of LDs (Extended Data Fig. 7e,f). To further investigate the effects of GNE-317 we performed RNA-seq of *APOE4/4* iMGs treated with fAβ in the presence or absence of the drug. GNE-317 reduced the expression of genes involved in lipid synthesis and reduced the genes indicative of a dysfunctional microglia state, such as inflammatory cytokine production and lysosomal accumulation (Extended Data Fig. 7g,h) and increased expression of genes involved in lipid degradation, microglial homeostasis and neuroprotective growth factors, such as *BDNF* and *FGF1* (Extended Data Fig. 7i,j and Supplementary Table 4). On GNE-317 treatment, genes in the PI3K/mTOR and autophagy pathways exhibit changes in expression and we also observed an increase in LC3B protein levels (Extended Data Fig. 7d,k–n). This may indicate that GNE-317 treatment reduced LD concentrations through increasing autophagy, a mechanism which is known to regulate LD concentrations^27–29^; however, these observations do not conclusively show increased autophagic flux after GNE-317 treatment.

## LD^+^ iMG induce pTau and apoptosis in neurons

**Fig. 4:**
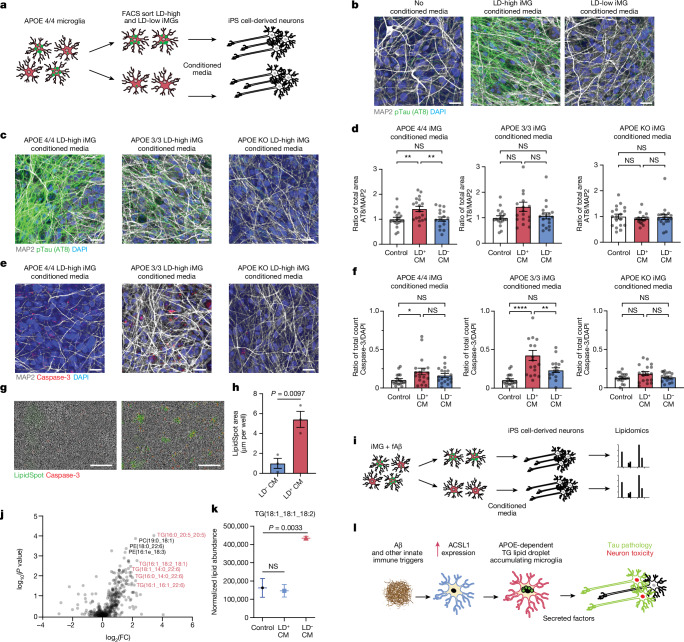
LD^+^ microglia induce Tau phosphorylation and apoptosis in neurons. **a**, Schematic of LDAM-specific conditioned media (CM) exposure to neurons. **b**, Immunofluorescence images of iPS cell-derived neurons exposed to no CM (left), LD^+^ *APOE4/4* iMG-CM (middle) and LD^−^ *APOE4/4* iMG-CM (left). Cells were stained for DAPI (blue), MAP2 (grey) and pTau (AT8, green). Scale bars, 20 μm. **c**, Immunofluorescence images of iPS cell-derived neurons exposed to LD^+^ *APOE4/4* iMG-CM (left), LD^+^ *APOE3/3* iMG-CM (middle), LD^+^ APOE-KO iMG-CM (right). Cells were stained for DAPI (blue), MAP2 (grey) and pTau (AT8, green). Scale bars, 20 μm. Data replicated in at least two independent experiments. **d**, Quantification of images as presented in **c**. Each dot represents a random filed image (*n* = 18) across three replicate wells per condition; one-way ANOVA; mean ± s.e.m. **e**, Immunofluorescence images of iPS cell-derived neurons under conditions in **c** stained for DAPI (blue), MAP2 (grey) and cleaved caspase-3 (red). Scale bars, 20 μm. **f**, Quantification of images as presented in **e**. Each dot represents a random filed image (*n* = 18) across three replicate wells per condition; one-way ANOVA; mean ± s.e.m. **g**, Images of neurons exposed to LD^−^ *APOE4/4* iMG-CM (left) and LD^+^ *APOE4/4* iMG-CM (right). Cells were stained with LipidSpot (green) and activated caspase-3 dye (red). Scale bars, 200 μm. **h**, Quantification LipidSpot fluorescence (*n* = 4 replicate wells per condition; two-sided *t*-test; mean ± s.e.m). **i**, Schematic of lipidomics experimental design of neurons treated with CM. **j**, Volcano plot representing lipids detected in neurons after treatment of LD^+^ iMG-CMa versus LD^−^ iMG-CM. Triglyceride species are highlighted in red. **k**, Lipidomic measurements of one lipid species detected in lipidomic analysis. Individual dots represent replicate wells (*n* = 3 replicate wells per condition; one-way ANOVA; mean ± s.e.m). **l**, Schematic of the proposed role of LD^+^ microglia in neurodegeneration. *P < 0.01, **P < 0.001, ****P < 0.0001.

To investigate the effect of LDAM on neurons, *APOE4/4* iMG were FACS sorted into LD-high (top 10% BODIPY signal) and LD-low fractions (bottom 10% BODIPY signal) and cultured for 12 h in neurobasal media to create conditioned media. *APOE4/4* iPS cell-derived human neurons were then grown in complete media containing 10% of the LD-high or LD-low *APOE4/4* iMG-conditioned media, as well as an untreated control condition (Fig. 4a). This approach takes inspiration from recent work showing the deleterious effects of conditioned media on neurons from astrocytes^30^ and microglia^4^. To investigate whether LDAM-specific conditioned media induced hallmarks of AD pathology, the human iPS cell-derived neurons were then stained with monoclonal antibody AT8 to detect phosphorylated Tau (pTau). Only the LD-high iMG-conditioned media induced high concentrations of pTau in the iPS cell-derived neurons, whereas the LD-low iMG-conditioned media induced similar concentrations of pTau in the iPS cell-derived neurons as the untreated condition (Fig. 4b). This effect was similar when conditioned media from *APOE3/3* and *APOE4/4* iPS cell-derived iMG were used to treat the human neurons but absent when conditioned media from *APOE*-KO iMGs were used (Fig. 4c,d). Likewise, conditioned media from *APOE3/3* and *APOE4/4* iMGs with a higher concentrations of LDs induced caspase activation in human neurons, whereas conditioned media from *APOE*-KO iMG had no effect (Fig. 4e,f).

**Extended Data Fig. 8:**
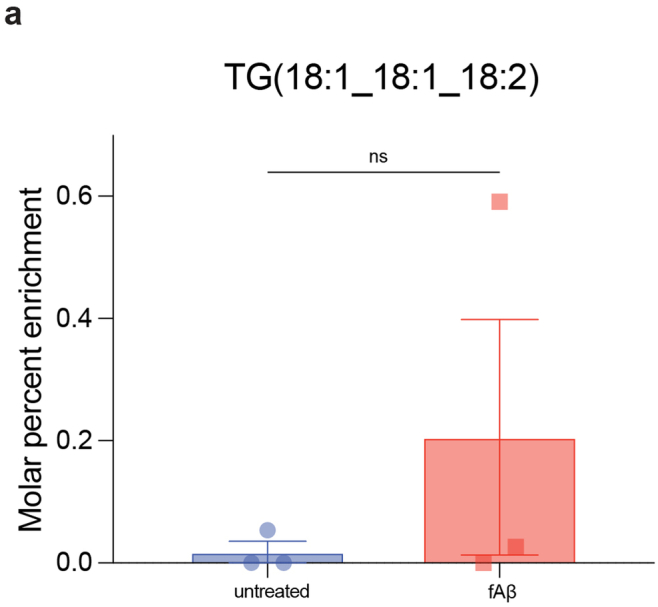
Detection of triglyceride synthesized in microglia taken up by neurons through labelled ^13^C-glucose tracing. **a**, Measurement of ^13^C-labelled triglycerides synthesized in microglia and profiled in neurons by lipidomics after exposure to microglia conditioned media. Microglia grown in uniformly labelled ^13^C-glucose (U-13C6-glucose) were challenged with fAβ or untreated. Each dot represents an individual replicate. n = 3 wells per condition; unpaired t-test; mean ± s.e.m.).

Intriguingly, human neurons treated with the LD-high conditioned media showed an increase in LipidSpot staining (Fig. 4g,h). To investigate which lipids accumulate in iPS cell-derived neurons on LDAM conditioned media treatment, we performed lipidomics on neurons exposed to LD-high and LD-low conditioned media. The iPS cell-derived neurons exposed to LD-high conditioned media contained higher concentrations of the triglyceride lipid species which accumulate in iMG on fAβ challenge (Fig. 4i–k and Supplementary Table 7). ^13^C-labelled TAG lipids synthesized in microglia were also observed in mouse neurons treated with conditioned media from BV2s grown in ^13^C-labelled glucose, however, this was not robustly detected across several replicates perhaps due to the low isotopic enrichment of these lipids (Extended Data Fig. 8a).

## Discussion

Here, we report a microglial state present in human brains defined by the accumulation of lipids with concomitant upregulation of genes involved in lipid synthesis; these cells are phenotypically similar to previously reported mouse LDAM. We report that LDAM are more prevalent in AD brains compared to controls and enriched in individuals with the *APOE4/4* genotype. This finding is confirmed by immunofluorescence for *ACSL1*, a key regulator of LD biogenesis, which may serve as a useful functional protein marker of human LDAM. We also describe the induction of triglyceride synthesis and LD accumulation following fAβ treatment in iMG and that the LD induction is greater in isogenic *APOE4/4* iMG than *APOE3/3* iMG. Conditioned media from LD-high microglia induce Tau phosphorylation and neurotoxicity in an APOE-dependent manner. Exposure of neurons to LD-high conditioned media increased the concentrations of the same triglyceride species observed to accumulate in microglia. Because neurons in AD do not seem to upregulate *ACSL1* in AD (Fig. 1f), we speculate that lipids accumulating in neurons are derived from microglia. This opens the possibility for a new hypothesis for LDAM-mediated pathogenesis in AD, wherein Aβ induces microglial triglyceride lipid synthesis, LD accumulation and subsequent secretion of neurotoxic factors in an APOE-dependent manner. In this model, these lipids might be transferred to neurons thereby inducing the hallmarks of neurodegeneration (Fig. 4l).

Whereas recent studies have focused on the role of *APOE* in astrocytes^31^, endothelial cells^32^, neurons^33^ and oligodendrocytes^34^, the LDAM expression signature, such as *ACSL1* and *NAMPT* upregulation, seems to be unique to microglia. It is likely that the *APOE* genotype contributes to AD pathogenesis through several distinct mechanisms unique to individual cell-type dysfunction or through crosstalk between these cell types. For example, cell culture models have demonstrated that hyperactive neurons can release *APOE*-associated fatty acids which are taken up by astrocytes and buffer lipotoxic stress^35^. It is possible that AD-associated *APOE* variants modify this neuroprotective transfer of lipids from hyperactive neurons to astrocytes. Astrocytes may also play a role in buffering the effects of the LDAM-secreted factors we describe here. Further studies are required to better understand the intercellular crosstalk of lipids across various central nervous system cell types in the context of AD-associated *APOE* variants.

A recent report showed that innate immune triggers (for example, *Escherichia coli* and *Salmonella*) induce LD formation in peripheral macrophages as part of an evolutionarily conserved antimicrobial defence in which LDs coated with antimicrobial proteins, such as cathelicidin (CAMP), kill bacteria^8^. We speculate that a similar programme can be triggered in human microglia exposed to Aβ, LPS and other innate immune activators and disrupt brain homeostasis. Protein aggregates found in other neurodegenerative diseases may trigger the LDAM state. For example, alpha-synuclein binding to TLR2 and TLR5 induces microglial NLRP3 inflammasome activation, which is a shared signature seen in LDAM^36^. Given that we recently identified that LDAM are abundant in the ageing mouse brain, LDAMs may also be triggered by hitherto unknown protein aggregates and innate immune activators which accumulate with age. Interestingly, the most enriched pathway in human LD-containing iMGs is ‘cellular senescence’, similar to lipid-laden ‘foamy macrophages’ in atherosclerosis which have a senescent phenotype and are drivers of pathology^37^. Perhaps in the natural ageing of various organs, LD-accumulating tissue-resident macrophages represent a general class of senescent myeloid cells which are drivers of tissue inflammation.

One strategy to clear LD accumulation in microglia, which we present here, is PI3K inhibition, shown to increase autophagy^38^. Activation of innate immune receptors such as TLR4 in microglia suppresses autophagy^39^ and this mechanism has been shown to increase LD concentrations in microglia^40^. Here, we report changes in RNA and protein concentrations of autophagy-associated genes and decreased LDs on PI3K inhibition in human microglia. However, these experiments do not conclusively demonstrate that there is increased autophagic flux of lipids in this context and further mechanistic experiments are required to resolve this uncertainty. Enhancing autophagy has previously been explored as a strategy to modulate neurodegeneration disease progression but with a focus on autophagic degradation of proteins^41^, as opposed to the autophagic degradation of intracellular LDs (lipophagy). Future investigation into the beneficial role of specifically increasing microglial lipophagy in AD models may better elucidate the damaging roles LDAM may have in AD.

In summary, we discovered that the *APOE4* genotype facilitates the microglial transition to an evolutionarily conserved, maladaptive and damaging LDAM state in response to innate immune triggers including Aβ. Future studies will have to determine whether protective *APOE* variants operate as antagonists to this microglial LDAM transition by limiting LD accumulation.

## Methods

### Single-nucleus RNA sequencing of human brain tissue

Frozen superior frontal gyrus and fusiform gyrus tissue blocks and pathology clinical reports were obtained from the Banner Sun Health Research Institute Brain and Body Donation Program in accordance with institutional review boards and policies at both Stanford School of Medicine and Banner Sun Health Research Institute. All samples obtained from Banner Sun Health Research Institute were stored at −80 °C until the time of processing. Isolation of nuclei from frozen brain tissue: 20–50 mg of flash-frozen human brain tissue isolated from the frontal cortex was thawed in 2 ml of ice-cold homogenization buffer (molecular biology grade water, 260 mM sucrose, 30 mM KCl, 10 mM MgCl, 20 mM tricine-KOH, 1 mM dithiothreitol, 500 μM spermidine, 150 μM spermine, 0.3% NP-40, protease inhibitor, RNAse inhibitor) for 5 min in a prechilled dounce homogenizer. Tissue was dounced with ‘A’ loose pestle for 10 strokes then dounced by 20 strokes with ‘B’ tight pestle. The resulting homogenate was passed through a 70 μm Flowmi strainer into a prechilled 1.5 ml tube. Nuclei were pelleted by centrifugation for 5 min at 4 °C at 350 relative centrifugal force in a fixed-angle centrifuge. All but 50 μl of supernatant (containing cytosolic RNA) was removed by pipetting and nuclei were resuspended in 1× homogenization buffer to a total volume of 400 μl and gently mixed by pipetting and transferred to a prechilled 2 ml protein-lobind tube. A total 400 μl of 50% iodixanol solution (OptiPrep Sigma catalogue no. D1556) was gently mixed by pipetting with 400 μl of resuspended nuclei to make a 25% iodixanol solution. A total 600 μl of a 30% iodixanol solution was gently layered underneath the 25% iodixanol solution containing isolated nuclei, without mixing iodixanol layers. Next, 600 μl of a 40% iodixanol solution was gently layered underneath the 30% iodixanol solution, without mixing iodixanol layers, resulting in a total volume of 2 ml. Samples were centrifuged in a prechilled swinging-bucket centrifuge for 20 min at 4 °C at 3,000 relative centrifugal force with the brake off. After centrifugation, the top 1 ml of the iodixanol layer (25% solution containing myelin and larger debris) was aspirated using a vacuum down to 1 ml total volume, containing the nuclei band. Nuclei were isolated from smaller cellular debris by removal by pipetting of the top 200 μl of the nuclei layer (at a volume between 800 μl and 1 ml in the 30%–40% iodixanol interface). A total 200 μl of isolated nuclei were diluted in 200 μl nuclei wash buffer (phosphate-buffered saline (PBS) with 0.1% BSA and 0.2 U μl^−1^ of RNase inhibitor. The snRNA-seq libraries were prepared from nuclei using the Chromium Next GEM Single Cell 3′ v.3.1 according to the manufacturer’s protocol (10x Genomics). Nuclei were counted using with a TC20 Automated Cell Counter (Bio-Rad) and loaded into a 10× droplet generator at a concentration of 8,000 nuclei per sample. Thirteen polymerase chain reaction (PCR) cycles were applied to generate complementary DNA and 15 cycles for final library generation. The final snRNA-seq libraries were sequenced on a NovaSeq 6000.

### snRNA-seq quality control

Raw gene counts were obtained by aligning reads to the hg38 genome (refdata-gex-GRCh38-2020-A) using CellRanger software (v.4.0.0) (10x Genomics). To account for unspliced nuclear transcripts, reads mapping to pre-messenger RNA were also counted. All subsequent analysis was implemented in Python (v.3.9.12) based on the Scanpy^42^ (v.1.9.1) single-cell data analysis package, except where stated otherwise. Count data were first screened for doublets with the Scrublet (v.0.2.3) Python package^43^. Once each cell was doublet scored, we applied a separate doublet score threshold per sample to discard doublets from the data. Thresholds were identified between 0.15 and 0.5 per sample on the basis of the sample-wise doublet score histograms (Extended Data Fig. 1a). We then applied standard filtering rules following the guideline of ref. ^44^. We used the Scanpy (v.1.9.1) package to discard cells with (1) fewer than 500 genes or (2) less than total 1,000 reads or (3) more than 10% mitochondrial reads or (4) more than 10% ribosomal reads. Counts were then counts-per-million (c.p.m). scaled and log-normalized for downstream analysis.

### Global data integration and clustering

We merged all data across the samples and used standard methods of Scanpy (v.1.9.1) to select the top 2,500 highly variable genes and calculate the top 20 principal components. The previous number of principal components were identified with the elbow method. We then used the Python implementation of BBKNN (v.1.5.1), a fast batch correction algorithm suitable for large datasets to integrate data across the samples^45^. BBKNN calculates a batch-corrected neighbourhood graph from the imputed principal components. We set the individual sample IDs as batch labels to correct for potential sample-wise batch effects. We then used the batch-corrected neighbourhood graph to run Leiden clustering^46^ and to calculate a global UMAP embedding with default parameters in Scanpy (v.1.9.1). We used these embeddings to annotate the cells. Note that BBKNN does not modify the count data in any ways but returns a neighbourhood graph which we use for the noted downstream analyses.

### Cell-type annotation

Leiden clusters were annotated one-by-one on the basis of domain-specific expertise. We investigated each Leiden cluster separately on the basis of the expression of common cell-type markers (excitatory neurons, *Slc17a7*; inhibitory neurons, *Gad1*; oligodendrocytes, *Mog*; endothelial cells, *Cldn5*; astrocytes, *Aqp4*; microglia, *Cd74*) and annotated the clusters accordingly. Marker gene expression analysis was implemented in Python (v.3.9.12) and Scanpy (v.1.9.1).

### Microglia pseudobulk differential gene expression

We summed the raw counts per patient sample and hence derived ‘pseudobulk’ samples^47^ from the single-cell counts. We then used Deseq2 (ref. ^48^) to perform bulk data normalization and differential gene expression (DGE) in R (v.4.3). We followed the standard Deseq2 analysis steps and conducted sequencing depth based count normalization across all APOE4/4, APOE3/3 and control samples. Then, we performed DGE analysis at standard parameters. The resulting log-scaled fold changes were shrunken using the standard ‘apeglm’ approach. In every comparison, we discarded genes used for QC filtering (Rb* and Mt-*) from the DGE analysis as these may be biased by the quality-control process. *P* values were corrected with Benjamini–Hochberg procedure^49^ (false discovery rate (FDR) = 0.05) per comparison.

### Microglia subsampling

To conduct unbiased single-cell level downstream analysis of the microglial cells which is not biased towards any of the patient samples, first we subsampled the microglia cluster with purification^50^. We used the first 20 principal components calculated on the basis of the top 2,000 highly variable genes as input and selected 1,000 cells per individual group (control, AD-APOE3/3 and AD-APOE4/4).

### Single-cell differential gene expression

We performed DGE analysis on the subsampled data with MAST^51^ in R (v.4.3) by using the Seurat package. We set gender, expired age, postmortem interval and the percentage of mitochondrial and ribosomal counts as covariates. We performed (1) pairwise comparisons (three in total) across the three individual groups (control, AD-APOE3/3 and AD-APOE4/4) and (2) ‘one-versus-rest’ comparisons between the three annotated microglial states (HOMEOSTATIC, DAM and LDAM). *P* values were corrected with Benjamini–Hochberg procedure^49^ (FDR = 0.05) per comparison.

### Microglia subclustering

Subsampled microglia data were normalized as described previously^3,52^. Briefly, starting from the raw counts, gender, expired age, postmortem interval and the percentage of mitochondrial/ribosomal counts were first regressed out with the regress_out function of the scanpy package (v.1.9.1). We then used the first 20 principal components based on the top 2,000 highly variable genes as input to repeat the Leiden clustering and UMAP visualization steps with default parameters in Scanpy (v.1.9.1) on this corrected microglia subcluster data. To characterize each subcluster, we calculated the mean expression level of marker genes (HOMEOSTATIC—P2RY12, P2RY13, CX3CR1, TMEM119; DAM—CD9, ITGAX, CLEC7A, CD63, SPP1, LPL, TREM2, APOE; LDAM—NAMPT, ACSL1, DPYD, CD163). We then calculated signature scores by averaging the minimum–maximum normalized expression values of these per cell. We used these signature scores to annotate the subclusters and also mapped them to the UMAP space and scanpy performed kernel density estimation to locate them in the UMAP space (the single-cell landscape).

### Prefrontal cortex immunohistochemistry and immunofluorescence

Adjacent tissue processed for snRNA-seq was subjected to immunohistochemistry and immunofluorescence. Prefrontal cortex from each individual were cut with a razor blade and directly submerged in 4% paraformaldehyde at 4 °C for 24 h. They were then transferred to a 30% sucrose solution in 1× PBS and stored at 4 °C until the tissue sank in its vial. Tissues were then frozen in OCT compound, stored at −80 °C and sectioned on a cryostat (Leica CM3050S). For Oil Red O staining, slides with 10 μm sections of prefrontal cortex were removed from the −80 °C freezer and brought to room temperature for 5 min. The slides were first incubated in propylene glycol for 5 min and then Oil Red O solution from an Abcam Oil Red O stain kit (ab150678) for 2 h at room temperature. The sections were differentiated in 85% propylene glycol in distilled water for 1 min, incubated in haematoxylin for 1 min and rinsed with several changes of distilled water. The slides were sealed with a glass cover slide and ProLong Gold mounting media. Imaging was performed with a ZEISS Axioskop 2 Plus microscope. Oil Red O counts per image were quantified in ImageJ and we performed statistical analysis in Prism9 (Graphpad). For immunofluorescence, free-floating 50 μm sections were washed three times in PBST followed by blocking with 5% donkey serum in PBST for 1 h. Sections were incubated in PBS with 3% donkey serum and the following primary antibodies for 72 h at 4 °C: goat anti-Iba1 (1:500; Abcam, ab5076), rabbit anti-ACSL1 (1:100; Thermo Fisher PA5-78713) and mouse anti-β-amyloid (1:500; Cell Signaling Technologies, no. 15126). After primary antibody incubation, sections were washed three times in PBST and incubated in PBS and the following secondary antibodies for 2 h at room temperature: donkey anti-goat Alexa Fluor 488, donkey anti-rabbit Alexa Fluor 555 and donkey anti-mouse Alexa Fluor 647 (all 1:500; Invitrogen). Sections were incubated in DAPI (1:2,000; Thermo Fisher) for 10 min, then washed three times with PBST. Sections were mounted on microscope slides with ProLong Glass mounting media. Imaging was performed with a ZEISS LSM 900 confocal microscope ZEN 3.0 (Blue Edition) software.

### Immunofluorescence of mouse brain sections

Human APOE3-KI and APOE4-KI mice were cross-bred with mice overexpressing mutant human APP (J20 line) to generate J20/APOE4-KI and J20/APOE3-KI mice, as we reported previously^15^. All mouse lines were maintained on a C57Bl/6 J background. Sex- and age-matched wild-type mice were used as controls. Brains were collected from female J20/APOE4-KI and J20/APOE3-KI mice (*n* = 3 for each group) at 13 months of age. Brain sections were collected (30 μm) from paraformaldehyde-fixed right hemibrains on a sliding microtome fitted with a freezing stage as described previously. Free-floating 30 μm sections were washed three times in PBS followed by blocking with 10% donkey serum in PBS for 1 h. Sections were incubated in PBS with 5% donkey serum and Iba1 primary antibody (1:500; Wako 019-19741) for 72 h at 4 °C. After primary antibody incubation, sections were washed once in PBS and incubated in the following secondary antibody and LD dye for 2 h at room temperature: donkey anti-rabbit 555 (1:500; Invitrogen) and LipidSpot 488 (1:1000; Biotium). Sections were incubated in DAPI (1:2,000; Thermo Fisher) for 10 min, then washed three times with PBS. Sections were mounted on microscope slides with ProLong Glass mounting media. Imaging was performed with a ZEISS LSM 900 confocal microscope ZEN 3.0 (Blue Edition) software. All animal care and procedures complied with the Animal Welfare Act and were in accordance with institutional guidelines and approved by the institutional administrative panel of laboratory animal care at Stanford University.

### iPS cell maintenance and differentiation to microglia

Isogenic *APOE4/4* and *APOE3/3* iPS cells were generated as previously described^16^. iPS cells were maintained in StemFlex medium (Gibco, A3349401) and routinely passaged as clumps onto Matrigel(Corning, 40230)-coated plates. Differentiation into microglia was performed as previously described^17–19^. The iPS cells were first differentiated into haematopoietic progenitor cells (HPCs) following the manufacturer’s instructions using the commercially available STEMdiff Hematopoietic Kit (Stemcell Technologies, 05310). HPCs in suspension were transferred to plates containing an adherent layer of confluent primary human astrocytes (Thermo Fisher, N7805100), in media containing Iscove’s Modified Dulbecco’s Medium (Thermo Fisher), fetal bovine serum (FBS) and penicillin–streptomycin and 20 ng ml^−1^ of each of IL-3, GM-CSF and M-CSF (PeproTech) for 10 days. The iMG were harvested and transferred into homeostatic culture conditions adapted from ref. ^19^ (MGdM media) on Matrigel(Corning, 40230)-coated plates for 5–15 days before assay. For assays involving immunofluorescence for microscopy iMG were plated in homeostatic culture conditions (MGdM media) on fibronectin(StemCell, 07159)-coated plates to enhance cell adherence. All assays were performed under serum-free conditions (MGdM media).

### iMicroglia and macrophages immunofluorescence and live cell microscopy

iMicroglia or human macrophages were fixed in 4% paraformaldehyde for 10 min. Cells were washed with PBS followed by blocking with 5% donkey serum in PBS for 1 h. Cells were incubated in PBS with 3% donkey serum and the following primary antibodies overnight at 4 °C: rabbit anti-Perilipin 2 (1:200; Proteintech 15294-1-AP), rabbit anti-ACSL1 (1:100; Thermo Fisher PA5-78713), LC3B (1:300, Thermo Fisher Scientific, PA1-46286) and mouse anti-β-amyloid (1:500; Cell Signaling Technologies, no. 15126). After primary antibody incubation, cells were washed with PBS and incubated in PBS and the following secondary antibodies for 2 h at room temperature: donkey anti-rabbit Alexa Fluor 488, donkey anti-rabbit Alexa Fluor 555 and donkey anti-mouse Alexa Fluor 647 (all 1:500; Invitrogen). Sections were incubated in DAPI (1:2,000; Thermo Fisher) for 10 min, then washed three times with PBS. Cells grown and stained on coverslips were then mounted on glass microscope slides with ProLong Glass mounting media. Imaging was performed with a ZEISS LSM 900 confocal microscope ZEN 3.0 (Blue Edition) software. Additionally, cells were fixed in replicate wells of a 96-well plate and were imaged and quantified with Incucyte S3 analysis system (Essen Bioscience). For live cell microscopy, cells were untreated or treated with 5 μM Aβ fibrils or 10 μM GNE-317 (Selleckchem, S7798) for 24 h. After 24 h, media were changed and cells were incubated with LipidSpot 488 (biotium, 70065-T) in combination with pHrodo Red Zymosan Bioparticles (P35364, Thermo Fisher) or Lysotracker (L12492, Thermo Fisher), according to manufacturer’s instructions. For Aβ fibril formation, monomeric HFIP-treated Aβ protein (1–42) (Bachem catalogue no. 4090148) was formed into fibrils as previously described^53^. Four phase-contrast, green and red fluorescent images per well were acquired every 1 h for 36 h using an Incucyte S3 analysis system (Essen Bioscience). For each time point, red and green fluorescence was normalized to the phase confluence per well.

### Primary human macrophage cell culture conditions

Whole blood was obtained from Stanford Blood Center. Peripheral blood mononuclear cells isolation was performed by diluting sample in PBS (equivalent blood volume) and transferred on top of the Ficoll layer (GE Healthcare catalogue no. 17-5442-02). The tubes were then centrifuged at 400*g*, at room temperature, acceleration (slow) and brake (slow) for 30 min. After centrifugation, the upper layer was discarded and the peripheral blood mononuclear cells layer at the interphase was collected in a fresh 50 ml Falcon tube. The cells were washed twice with PBS and counted. Monocytes were then isolated using the Pan Monocyte Isolation Kit (Miltenyi Biotec catalogue no. 130-096-537) according to manufacturer’s instructions. Monocytes were cultured in X-VIVO 15 serum-free media (Lonza) and differentiated into macrophages with 20 ng ml^−1^ of M-CSF (Peprotech).

### BV2 cell culture conditions

Cells from the mouse microglial BV2 cell line were originally obtained from Banca Biologica e Cell Factory, IRCCS Azienda Ospedaliera Universitaria San Martino, Italy. Cells were maintained in DMEM (Life Technologies) supplemented with 5% FBS and antibiotics (penicillin 100 U ml^−1^, streptomycin 100 U ml^−1^ (Gibco), 10 mM glutamax (Gibco) under standard culture conditions (95% relative humidity with 5% CO_2_ at 37 °C). Adherent cells were split using 1× TrypLE (Gibco). Experiments with deuterated lipids were conducted by replacing glucose with 10 mM d-glucose (U-13C6, Cambridge isotope laboratories CLM-1396-PK) for 24 h before treatment with 5 μM Aβ fibrils or no treatment.

### Primary rat microglia cell culture conditions

Primary rat microglia were cultured as previously described^54^. P10-20 rats were transcardially perfused with ice-cold PBS and, immediately after perfusion, brains were rapidly dissected and placed into ice-cold PBS. Brain material was minced and transferred to an ice-cold dounce homogenizer (Wheaton) with ice-cold PBS containing 200 μl of 0.4% DNaseI per 50 ml of PBS. Tissue chunks were subjected to three successive rounds of three to ten gentle strokes of the homogenizer piston and centrifuged. Percoll PLUS was added during centrifugation to separate myelin from cells. The cell suspension volume was adjusted to 33.4 ml with PBS and 10 ml of 100% isotonic Percoll (9 ml of Percoll PLUS (GE Healthcare), 1 ml of 10× PBS without Ca and Mg, 9 μl of 1 M CaCl_2_, 5 μl of 1 M MgCl_2_) was added and thoroughly mixed (23% isotonic Percoll final). Suspensions were centrifuged (15 min, 500*g*, 4 °C) and the supernatant and top layer of myelin were discarded. Rat CD11b/c (Microglia) MicroBeads (Miltenyi, catalogue no. 130-105-643) were used to enrich rat microglia using MACS columns according to manufacturer’s instructions. Serum-free rat microglia basal microglial growth medium was sterile-filtered and stored at 4 °C for up to 1 month and was comprised of: DMEM/F12 containing 100 U ml^−1^ of penicillin, 100 μg ml^−1^ of streptomycin, 2 mM glutamine, 5 μg ml^−1^ of *N*-acetyl cysteine, 5 μg ml^−1^ of insulin, 100 μg ml^−1^ of apo-transferrin and 100 ng ml^−1^ of sodium selenite, ovine wool cholesterol (1.5 μg ml^−1^, Avanti Polar Lipids), heparan sulfate (1 μg ml^−1^, Galen Laboratory Supplies). The final medium was comprised of basal media containing human TGFβ2 (2 ng ml^−1^, Peprotech), mouse IL-34 (100 ng ml^−1^, R&D Systems). Cells were plated on 24-well plates coated with poly-d-lysine (Gibco). Cells were grown in a humidified incubator held at 37 °C and 10% CO_2_. The 50% medium changes were performed every 2–3 days. For live cell microscopy, cells were untreated or treated with 5 μM Aβ fibrils (as described above) pre-incubated with an Aβ antibody (Cell Signaling Technologies, no. 15126) conjugated to an Alexa Fluor 555 secondary (Invitorogen), along with LipidSpot 488 (Biotium, 70065-T) and imaged for 24 h. All animal care and procedures complied with the Animal Welfare Act and were in accordance with institutional guidelines and approved by the institutional administrative panel of laboratory animal care at Stanford University.

### Preparation of slides for iPS cell-derived cultures for CARS imaging

The cell cultures, adherent to circular cover glasses, were positioned on secondary no. 1.5H cover glasses (Thorlabs) with a thin layer of PBS. To circumvent interference with lipid CARS signal generation, neither mounting media, antifade agents nor surfactants were used. The edges of the cover glass were sealed with VALAP, a homogeneous blend of equal parts petroleum jelly, lanolin and paraffin, which served to prevent sample desiccation.

### Characterization by CARS and confocal fluorescence microscopy

Intracellular lipids in iPS cell-derived cells were visualized and quantified using coherent anti-Stokes Raman scattering (CARS) microscopy. An inverted microscope (Nikon, Ti2-E equipped with a C2 confocal scanning head and a Nikon CFI Apochromat TIRF 100XC oil immersion objective) was used for this purpose. The C2 scanner was retrofitted with a slidable mirror (Optique Peter), allowing for a convenient switch between fluorescence excitation using the laser diodes (at wavelengths 405, 488, 561 and 647 nm) and CARS excitation. In CARS imaging mode, carbon–hydrogen (C–H) vibrations were coherently driven by temporally and spatially overlapping two near-infrared laser beams, generated by a picosecond-pulsed laser system (APE America, picoEmerald S with 2 ps pulse length, 80 MHz repetition rate and 10 cm^−1^ bandwidth) consisting of a 1,031 nm mode-locked ytterbium fibre laser and an optical parametric oscillator (OPO) tunable between 700 and 960 nm (pumped by the second harmonic of the 1,031 nm laser). The OPO wavelength was set to 797 nm to drive the symmetric stretching vibration of CH_2_ at 2,850 cm^−1^. The quadratic dependence of the CARS signal on the number density of the probed C–H vibrational group rendered sharp contrast for the lipid-dense regions without requiring external labels or disruptive sample preparations. The CARS signal generated by simultaneously scanning the two excitation beams over the sample was detected pixel-by-pixel with a photomultiplier tube (Hamamatsu, R6357) in the forward direction with optical filters which minimized background signals (Semrock; two FF01-640-20 and one FF01-750/SP). The excitation powers at the sample position were 18 mW for the pump (OPO) beam and 15 mW for the Stokes (1,031 nm) beam. For the iMG-like cells, 5–16 image stacks were acquired for each APOE genotype and Aβ treatment condition. Each stack comprised 19 slices at a resolution of 1,024 × 1,024 pixels (77.14 × 77.14 μm^2^) per image with a dwell time of 10.8 μs per pixel. Slices were spaced 0.4 μm apart, yielding a total imaging depth of 7.2 μm. Cell-specific immunohistochemistry facilitated the identification of the microglia (IBA1) with confocal fluorescence, which was collected in the same positions as the nonlinear imaging with a dwell time of 5.3 μs per pixel. The microglia were stained with Alexa Fluor 488. For selected APOE/treatment combinations, a CARS spectrum was acquired in the C–H stretching region for one field of view by varying the OPO wavelength between 785.5 and 801 nm, with 0.5 nm intervals per acquisition, amounting to a total of 32 image stacks making up the spectrum. All images were analysed using the Fiji distribution package of ImageJ. The CARS stacks underwent east shadows correction, Gaussian blur filtering (*σ* = 1) and background subtraction (30 pixel rolling ball radius). Lipid particles were identified by thresholding using the FindFoci method, with a search parameter for half peak value of 0.1, peak parameter for minimum peak height relative to background of 0 and a minimum peak size of 200 pixels. Cells were identified by thresholding with the Triangle method. Intracellular lipid features were quantified using the ‘3D Objects Counter’ command. To generate spectral line plots, the spectra from all individual intracellular lipid features in each cell were normalized and averaged.

### iMicroglia secreted cytokine/chemokine assay

APOE4/4 iMG were treated with 5 μM Aβ fibrils stained with LipidSpot 488 as described above then top 10% highest and 10% lowest LipidSpot fluorescent cells were seeded into 96-well plates at 5,000 cells per well by FACS (Sony, MA900) in 100 μl of MGdM media for 24 h. Media supernatant was collected and secreted signalling proteins were measured in culture supernatants by the Human Immune Monitoring Center at Stanford University using a Human 48-plex Luminex Procarta Immuno-assay (Thermo Fisher).

### ATAC-seq library preparation

Approximately 100,000 iMG were pelleted at 300*g* and 4 °C. Next, pellets were gently resuspended in ice-cold 50 μl of lysis buffer (10 mM Tris-HCl pH 7.4, 10 mM NaCl, 3 mM MgCl_2_, 0.1% IGEPAL CA-630) and spun down at 500*g* for 10 min and 4 °C. The supernatants were discarded and pellets gently resuspended in 50 μl of transposition reaction mix (25 μl of tagment DNA buffer (Nextera, Illumina), 2.5 μl of tagment DNA enzyme (Nextera, Illumina), 22.5 μl of nuclease free water) and incubated at 37 °C for 30 min. Tagmented DNA was purified using MinElute PCR purification kit (Qiagen) and size selected for 70–500 base pairs (bp) using AmpureXP beads (Beckman Coulter). Libraries were constructed and amplified using 1.25 μM Nextera index primers and NEBNext High-Fidelity 2× PCR Master Mix (New England BioLabs). A quantitative PCR was run to determine the optimal number of cycles. Libraries were gel size selected for 165–300 bp fragments and single-end sequenced for 100 cycles (PE100) on an Illumina NovaSeq 6000.

### ATAC-seq analysis

Bowtie2 with default parameters was used to map ATAC-seq. HOMER was used to convert aligned reads into ‘tag directories’ for further analysis^55^. ATAC-seq experiments were performed in replicate and peaks were called with parameters -L 0 -C 0 -fdr 0.9 -minDist 200 -size 200. Irreproducible discovery rate (IDR) was used to test for reproducibility between replicates. IDR peaks were merged using HOMER mergePeaks and annotated with HOMER annotatePeaks.pl with a size parameter of 250. Differential enhancer peaks (±3 kilobases from transcription start site) were identified using DESeq2 with FC > 1 and adjusted *P* < 0.05. HOMER motif analysis (findMotifsGenome.pl) including known default motifs and de novo motifs was used to identify motifs enriched in enhancer peak regions over background. The background sequences were from random GC-matched genome sequences. The UCSC genome browser was used to visualize ATAC-seq data.

### iMicroglia bulk RNA sequencing

For Aβ-treated conditions, iMG were exposed to 5 μM Aβ fibrils for 24 h or DMSO before RNA isolation. For LD-high versus LD-low conditions, iMG were exposed to 5 μM Aβ fibrils for 24 h, stained with LipidSpot 488 as described above and top 10% highest and 10% lowest LipidSpot fluorescent cells were separated by FACS (Sony, MA900). iMicroglia RNA was isolated from the cell pellets using an RNeasy Plus Micro kit (Qiagen, 74034). The mRNA was transcribed into full-length cDNA using a SMART-Seq v.4 Ultra Low Input RNA kit (Clontech) according to the manufacturer’s instructions. Full-length cDNA (150 pg) was processed using a Nextera XT DNA library preparation kit (Illumina) according to the manufacturer’s protocol. Library quality was verified using the Agilent 2100 Bioanalyzer and the Agilent High Sensitivity DNA kit. Sequencing was carried out using an Illumina NexSeq 550, paired-end, 2× 100 bp depth sequencer. Reads were mapped to the human hg38 reference genome using STAR (v.2.5.1b). Raw read counts were generated with STAR using the GeneCounts function. Differential expression in RNA-seq was analysed using the R package DESeq2 (ref. ^48^).

### Electron microscopy

Cells were grown on aclar and then fixed in Karnovsky’s fixative: 2% glutaraldehyde (EMS catalogue no. 16000) and 4% paraformaldehyde (EMS catalogue no. 15700) in 0.1 M sodium cacodylate (EMS catalogue no. 12300) pH 7.4 for 1 h, chilled and sent to Stanford Cell Sciences Imaging Facility on ice. They were then postfixed in cold 1% osmium tetroxide (EMS catalogue no. 19100) in water and allowed to warm for 2 h in a hood, washed 3× with ultrafiltered water, then bloc stained 2 h in 1% uranyl acetate at room temperature. Samples were then dehydrated in a series of ethanol washes for 10 min each at room temperature beginning at 30%, 50%, 70%, 95%, changed to 100% 2X, then propylene oxide for 10 min. Samples are infiltrated with EMbed-812 resin (EMS catalogue no. 14120) mixed 1:1 and 2:1 with propylene oxide for 2 h each. The samples are then placed into EMbed-812 for 2 h opened then placed into flat moulds with labels and fresh resin and placed into 65 °C oven overnight. Sections were taken around 90 nm, picked up on formvar/carbon-coated Cu grids, stained for 40 s in 3.5% uranyl acetate in 50% acetone followed by staining in Sato’s Lead for 2 min. These were observed in the JEOL JEM-1400 120 kV and photos were taken using a Gatan Orius 2k × 4k digital camera.

### GW lipid droplet CRISPR–Cas9 screen

U937 cells were acquired from the American Type Culture Collection (CRL-1593.2). Cells were maintained in suspension culture using spinner flasks for library propagation and tissue culture plates for single-gene knockout lines, all in sterile-filtered U937 growth medium (RPMI-1640 supplemented with 2 mM glutamine, 100 U ml^−1^ of penicillin, 100 mg ml^−1^ of streptomycin and 10% heat-inactivated FBS. Cells were cultured in a humidified 37 °C incubator set at 5% CO_2_. A ten-sgRNA-per-gene CRISPR–Cas9 deletion library (Human CRISPR knockout library was a gift from M. Bassik (Addgene no. 101926-101934)) was infected into Cas9-expressing U937 cells as described^56^. Briefly about 300 million U937 cells stably expressing SFFV-Cas9-BFP were infected with the ten-guide-per-gene genome-wide sgRNA library at a multiplicity of infection less than one. Infected cells underwent puromycin selection (1 μg ml^−1^) for 5 d, after which puromycin was removed and cells were resuspended in normal growth medium without puromycin. After selection, sgRNA infection was measured as more than 90% of cells as indicated by measuring mCherry-positive cells with flow cytometry. For the LD screen, the 10% FBS in the media was replaced with 10% aged (more than 80 years of age) human plasma pooled from several donors, dialysed and delipidated to better represent the aged circulating monocyte environment. U937s were then stained with BODIPY 493/503 (1:2,000 from 1 mg ml^−1^ of stock solution in DMSO; Thermo Fisher) and top 10% highest and 10% lowest BODIPY fluorescent cells were separated by FACS (Sony, MA900). Screens were performed in duplicate. At the end of each screen genomic DNA (gDNA) was extracted for all screen populations separately according to the protocol included with QIAGEN Blood Maxi Kit. Using known universal sequences present in the lentivirally incorporated DNA, sgRNA sequences were amplified and prepared for sequencing by two sequential PCR reactions as described^16^. Products were sequenced using an Illumina Nextseq to monitor library composition (30–40 million reads per library). Trimmed sequences were aligned to libraries using Bowtie, with zero mismatches tolerated and all alignments from multimapped reads included. Guide composition and comparisons across bound and unbound fractions were analysed using casTLE^57^ v.1.0. Enrichment of individual sgRNAs was then calculated as a median-normalized log ratio of the fraction of counts, as described^57^. For each gene, a maximum likelihood estimator was used to identify the most likely effect size and associated log-likelihood ratio (confidence score) by comparing the distribution of gene-targeting guides to a background of non-targeting and safe-targeting guides.

### iMG CRISPR–Cas9 screen

iMG CRISPR–Cas9 screens were performed using modified sgRNA lentiviral infection with Cas9 protein electroporation (SLICE) approach^58^. The human CRISPR knockout library was a gift from M. Bassik (Addgene no. 101927). In brief, about 40 million *APOE4/4* iPS cells were lentiviral infected with the ten-guide-per-gene sgRNA sublibraries at a multiplicity of infection less than one. Infected cells underwent puromycin selection (0.8 μg ml^−1^) for 2 d after which point puromycin was removed and cells were resuspended in normal growth media without puromycin. iPS cells were differentiated into iMG as described above. At day 15 of iMG–human astrocyte coculture, iMG cells were pelleted and resuspended in Lonza electroporation buffer P3 (Lonza, V4XP-3032) at 5 million cells per 100 ml. Cas9 protein (MacroLab, 40 mM stock) was added to the cell suspension at a 1:10 v/v ratio. Cells were electroporated at 5 million cells per 100 ml of cells per cuvette using a Lonza 4-D nucleofector with pulsecode DP-148 (Lonza, VVPA-1002). Cells were cocultured for five more days with human astrocyte before transferring cells to homeostatic culture conditions (MGdM media) as described above. In duplicate culture conditions, cells were treated with 5 μM Aβ fibrils for 24 h, stained with BODIPY 493/503 (1:2,000 from 1 mg ml^−1^ of stock solution in DMSO; Thermo Fisher) and top 10% highest and 10% lowest BODIPY fluorescent cells were separated by FACS (Sony, MA900). The gDNA was extracted for all populations separately using a QIAGEN Blood Midi Kit, sgRNA sequences were amplified by PCR using common flanking primers and indices and adaptors were attached to amplicons in a second PCR. Deep sequencing of sgRNA sequences on an Illumina Nextseq550 was used to monitor library composition. Guide composition was analysed and compared to the plasmid library and between conditions using casTLE^57^. The enrichment of individual guides was calculated as the log ratio between LD-high and LD-low populations and gene-level effects were calculated from ten guides targeting each gene. *P* values were then calculated by permutating the targeting guides as previously described^57^.

### Neuronal differentiation of hiPS cells

Human induced pluripotent stem (hiPS) cells were derived into neurons as previously described^16^, with slight modifications to increase yield. The hiPS cells were dissociated with Accutase followed by quenching with warm (37 °C) N2B27 medium. N2B27 medium consisted of 1:1 DMEM/F12 (11330032, Thermo Fisher) and Neurobasal Media (21103049, Thermo Fisher), 1% N2 Supplement (21103049, Thermo Fisher), 1% B27 (17504044, Thermo Fisher), 1% MEM non-essential amino acids (11140050, Thermo Fisher), 1% Glutamax (35050061, Thermo Fisher) and 0.5% penicillin–streptomycin (15140122, Thermo Fisher). Dissociated hiPS cells were then pelleted and resuspended in embryoid body media (10 µM SB431542 (1614, Tocris) and 0.25 µM LDN (04-0074, Stemgent) in N2B27) with 10 µM ROCK inhibitor (1254, Tocris), followed by growth in suspension in a T-75 flask (12-565-349, Fisher Scientific). For the first 3 h, the flasks were shaken manually once per hour. On days 2, 4 and 6, the media were replaced with fresh embryoid body medium without ROCK inhibitor. On day 8, spheres were plated as neural progenitors onto a 10 cm dish precoated with growth factor-reduced (GFR) Matrigel (CB-40230A, Fisher Scientific). Neural progenitors were allowed to form neuronal rosettes and sustained in N2B27 media alone for days 8–15. During this period, half of the media were replaced every 48–72 h, depending on confluency and media consumption. On day 16, the neuronal rosettes were lifted using STEMdiff Neural Rosette Selection Reagent (05832, StemCell Tech) and plated onto three wells of a six-well plate precoated with GFR Matrigel in N2B27 with 100 ng ml^−1^ of FGFb (100-18B, Peprotech) and 100 ng ml^−1^ of EGF (AF-100-15, Peptrotech). This N2B27 medium with FGFb and EGF was replaced daily. On day 20, the neural progenitors were passaged by dissociating with Accutase, quenching with N2B27 and resuspending in STEMdiff Neural Progenitor Medium (05833, StemCell Tech) at 1.2 × 10^6^ cells per 2 ml for one well of a six-well plate, precoated with GFR Matrigel. For days 21–27, neural progenitor cells were fed with fresh Neural Progenitor Medium every day. On day 28, the neural progenitor cells were dissociated with Accutase, N2B27 media was added to bring the volume of cell suspension to 40 ml and cells were filtered through a 40 µm cell strainer (08-771-1, Fisher). Cells were then collected by centrifugation and resuspended in complete neuronal medium. Neuronal media consisted of 10 ng ml^−1^ of BDNF (450-02, Peprotech) and 10 ng ml^−1^ of GDNF (450-10, Peprotech) in N2B27 with 10 nM DAPT (2634, Tocris). Next, the cells were counted and plated at a concentration of 2 × 10^5^ cells per well onto 12 mm coated glass coverslips (354087, Corning) in a 24-well plate. Coverslips were coated with poly-l-lysine (P4707, Sigma-Aldrich) and mouse-Laminin (23017015, Gibco) before plating. Then 50% of culture medium was replaced on maturing neurons every 3–4 days. DAPT was removed after the first week. Experiments were performed on neuronal cultures that had been differentiated for 4 weeks.

### LDAM media treatment of neurons

LDAM-high and LDAM-low conditioned media from 1 million *APOE4/4*, *APOE3/3* and APOE-KO iMG were prepared by exposing iMG to 5 μM fAβ for 24 h, washed 3× with PBS, stained with LipidSpot 488 as described above then top 10% highest and 10% lowest LipidSpot fluorescent cells were sorted by FACS (Sony, MA900) and grown in N2B27 medium (as described above) supplemented with 100 ng ml^−1^ of IL-34, 10 ng ml^−1^ of CSF1 and 10 ng ml^−1^ of TGFβ for 12 h. Conditioned media were collected by centrifuging media supernatant at 2,000*g* for 10 min to remove cell debris. The 10% of the total volume from the iMG-conditioned media was added to fresh N2B27 with 10 ng ml^−1^ of BDNF (450-02, Peprotech) and 10 ng ml^−1^ of GDNF (450-10, Peprotech) before treating neurons. To treat neurons with the various LDAM media, N2B27 neuronal media were completely removed from the cells and replaced with the iMG-conditioned media containing GDNF and BDNF. After 48 h, the neurons were fixed for immunocytochemistry.

### Immunocytochemistry, imaging and quantification

Neurons were washed with 1× DPBS (14080055, Gibco) and fixed in 4% paraformaldehyde for 15 min. The neurons were then washed three times for 5 min with 1× DPBS (14080055, Gibco) containing 0.1% Tween. Next, the neurons were permeabilized and blocked with a 1 h wash in 1× DPBS (14080055, Gibco) containing 10% Normal Donkey Serum (017000121, Jackson Immuno) and 0.5% Triton-X. Cells were then stained with primary antibodies overnight at 4 °C targeting the following proteins: MAP2 (PA1-10005, Thermo Fisher Scientific, 1:5,000), AT8 (MN1020, Thermo Fisher Scientific, 1:500) and caspase-3 (9661, Cell Signaling Technology, 1:500). The secondary antibodies were IgG conjugated to Alexa Fluor 488 (donkey anti-mouse, A-21202, Life Technologies Corporation, 1:1,000), Alexa Fluor 594 (donkey anti-rabbit, A-21207, Life Technologies Corporation, 1:1,000) and Alexa Fluor 647 (donkey anti-chicken, 703-605-155, Jackson Immuno, 1:500). Coverslips were mounted to microscope slides with VECTASHIELD Prolong Gold with DAPI (H-1200-10, Vector Labs). Images were taken with a FV3000 confocal laser scanning microscope (Olympus) at ×20 or ×40. Image analysis to quantify AT8 or caspase-3 positivity in hiPS cell-derived neuron stains was performed using custom macros written in the open-source Fiji (ImageJ) software. For all image analyses, a standard threshold value was chosen and automatically applied to each channel of each image before measurement. For quantification of AT8 immunofluorescence, the total area of AT8 immunofluorescence was normalized to the total area of MAP2 immunofluorescence. For quantification of caspase-3 immunofluorescence, the total count of caspase-3 positivity was normalized to the total count of DAPI positivity.

### Preparation of mouse cortical primary neurons

Newborn mouse pups on the first day after birth were humanely killed through decapitation and their cortices were carefully collected using microdissection techniques. The freshly obtained cortices were then rinsed with a dissection medium (Thermo Fisher Scientific, 14170161) before being subjected to tissue dissociation using scissors, 0.25% trypsin and pipette trituration. Cell strainers with a pore size of 70 µm were used to remove any remaining tissue fragments from the digested cortices. The dissociated neurons were then plated onto 24-well plates with coverslips coated with poly-l-lysine (Newcomer Supply, 1339 A) using minimum essential medium (Thermo Fisher Scientific, 21010046) supplemented with 10% inactivated fetal calf serum (Thermo Fisher Scientific, 10438026), 2 mM glutamine and penicillin and streptomycin (Thermo Fisher Scientific, SV30010). After 24 h of initial seeding, a complete medium change was performed using neurobasal medium (Thermo Fisher Scientific, 21103049) supplemented with B27 (Invitrogen, 17504044) and 2 mM GlutaMax (Thermo Fisher Scientific, 35050061). The primary neuronal cells were maintained at a temperature of 37 °C with a 5% CO_2_ environment. A detailed protocol is described in ref. ^59^.

### Lipid extraction and lipidomics

Dried lipids were reconstituted by a buffer consisting of 50 μl of ACN:IPA:water in a ratio of 13:6:1 (v/v/v). The mixture was then vigorously mixed using a vortex for a duration of 10 min at 4 °C. Afterward, the samples were centrifuged at maximum speed for 10 min at 4 °C. A volume of 45 μl of the supernatant was then carefully transferred into glass insert vials for further analysis using liquid chromatography–mass spectrometry.

Lipid profiling was conducted using an ID-X tribrid mass spectrometer equipped with a heated electrospray ionization probe. C18-based lipid separation was performed using an Ascentis Express C18 column coupled with a guard column. The mobile phases consisted of ammonium formate and formic acid dissolved in water, acetonitrile and 2-propanol. The mass spectrometer parameters, including temperatures, resolutions, voltages and gas settings, were optimized for lipid analysis. HCD fragmentation and data-dependent tandem mass spectrometry were used for comprehensive lipid identification. LipidSearch and Compound Discoverer software were used for unbiased differential analysis and lipid annotation. Metabolite abundance was accurately quantified using TraceFinder (Thermo Fisher Scientific). Mass tolerance of 5 ppm was applied for the extraction of ion chromatograms, ensuring accurate measurement of lipid concentrations. The full detail of the method can be found in ref. ^60^.

### Reporting summary

Further information on research design is available in the Nature Portfolio Reporting Summary linked to this article.

## Online content

Any methods, additional references, Nature Portfolio reporting summaries, source data, extended data, supplementary information, acknowledgements, peer review information; details of author contributions and competing interests; and statements of data and code availability are available at 10.1038/s41586-024-07185-7.

### Supplementary information

Reporting Summary

Supplementary Table 1 Metadata for brain tissue used in single-nucleus RNA-seq.

Supplementary Table 2 DEG and KEGG enrichment analyses of microglia from single-nucleus RNA-seq of brain tissue.

Supplementary Table 3 Genome-wide CRISPR-KO screen analysis for LD levels.

Supplementary Table 4 iMG bulk RNA-seq DEG analyses.

Supplementary Table 5 ATAC-seq analysis comparing LD^+^ and LD^−^ iMGs.

Supplementary Table 6 Drug target, kinase, phosphatase CRISPR-KO screen analysis comparing LD^+^ and LD^−^ iMGs.

Supplementary Table 7 Lipidomics analysis comparing neurons ± MG LD conditioned media.

## Data Availability

Raw snRNA-seq data, iMG RNA-seq and iMG ATAC-seq data are available in the Gene Expression Omnibus under accession code GSE254205.

## Code availability

Code used for downstream analyses of the raw snRNA-seq count matrices is available at GitHub: https://github.com/twclab/LDAM.
