# Supplementary material for: APOE4/4 is linked to damaging lipid droplets in Alzheimer’s disease microglia
Source: Nature. 2024 Mar 13;628(8006):154–61. doi: 10.1038/s41586-024-07185-7 (PMC10990924; doi:10.1038/s41586-024-07185-7)
Supplement: Supplementary file 1 — Reporting Summary [file 41586_2024_7185_MOESM1_ESM.pdf]

nature portfolio

Corresponding author(s): Michael S. Haney, Róbert Pálovics, Christy Nicole Munson, Chris Long, Patrik Johansson, Oscar Yip, Wentao Dong, Eshaan Rawat, Elizabeth West, Johannes CM Schlachetzki, Andy Tsai, Ian Hunter Guldner, Bhawika S. Lamichhane, Amanda Smith, Nicholas Schaum, Kruti Calcuttawala, Andrew Shin, Yung-Hua Wang,Chengzhong Wang, Nicole Koutsodendris, Geidy E Serrano, Thomas G Beach, Eric M Reiman, Christopher K Glass, Monther Abu-Remaileh, Annika Enejder, Yadong Huang, Tony Wyss-Coray.

Last updated by author(s): 12/29/2023

Reporting Summary

Nature Portfolio wishes to improve the reproducibility of the work that we publish. This form provides structure for consistency and transparency in reporting. For further information on Nature Portfolio policies, see our Editorial Policies and the Editorial Policy Checklist.

Statistics

For all statistical analyses, confirm that the following items are present in the figure legend, table legend, main text, or Methods section.

n/a	Confirmed	
☐	☒	The exact sample size (*n*) for each experimental group/condition, given as a discrete number and unit of measurement
☐	☒	A statement on whether measurements were taken from distinct samples or whether the same sample was measured repeatedly
☐	☒	The statistical test(s) used AND whether they are one- or two-sided<br>*Only common tests should be described solely by name; describe more complex techniques in the Methods section.*
☐	☒	A description of all covariates tested
☐	☒	A description of any assumptions or corrections, such as tests of normality and adjustment for multiple comparisons
☐	☒	A full description of the statistical parameters including central tendency (e.g. means) or other basic estimates (e.g. regression coefficient) AND variation (e.g. standard deviation) or associated estimates of uncertainty (e.g. confidence intervals)
☐	☒	For null hypothesis testing, the test statistic (e.g. *F*, *t*, *r*) with confidence intervals, effect sizes, degrees of freedom and *P* value noted<br>*Give P values as exact values whenever suitable.*
☒	☐	For Bayesian analysis, information on the choice of priors and Markov chain Monte Carlo settings
☒	☐	For hierarchical and complex designs, identification of the appropriate level for tests and full reporting of outcomes
☒	☐	Estimates of effect sizes (e.g. Cohen's *d*, Pearson's *r*), indicating how they were calculated

Our web collection on statistics for biologists contains articles on many of the points above.

Software and code

Policy information about availability of computer code

Data collection	Incucyte S3 (Essen), ZEN Black (Zeiss), Sony MA900 Cell Sorter Software Version 3.1.1
Data analysis	CellRanger (v.4.0.0) (10x Genomics), Python (3.9.12), Scanpy (1.9.1), Scrublet (0.2.3), FlowJo (Treestar, 10.9), Prism 9 (GraphPad), R (DESeq2, ggplot2, tidyverse), ImageJ(1.53), Enrichr, casTLE(1.0)

For manuscripts utilizing custom algorithms or software that are central to the research but not yet described in published literature, software must be made available to editors and reviewers. We strongly encourage code deposition in a community repository (e.g. GitHub). See the Nature Portfolio guidelines for submitting code & software for further information.

Data

Policy information about availability of data

All manuscripts must include a data availability statement. This statement should provide the following information, where applicable:

- Accession codes, unique identifiers, or web links for publicly available datasets
- A description of any restrictions on data availability
- For clinical datasets or third party data, please ensure that the statement adheres to our policy

Raw sequencing data is deposited in NCBI GEO under accession code GSE254205 . Analyzed Single nucleus RNA-seq data (Extended Data Table 2), CRISPR screen Data (Extended Data Table 3,4), Bulk RNA-seq Data and ATAC-seq Data (Extended Data Table 5,6), and lipidomics data (Extended Data Table) are provided.

Research involving human participants, their data, or biological material

Policy information about studies with human participants or human data. See also policy information about sex, gender (identity/presentation), and sexual orientation and race, ethnicity and racism.

Reporting on sex and gender	For the postmortem human brain tissue used in this study, an equal mixture of self-reported sexes was included in this study (See Supplementary Table 1).
Reporting on race, ethnicity, or other socially relevant groupings	Race, ethnicity, or other socially relevant groupings are not reported for the postmortem human brain tissue in this study.
Population characteristics	Postmortem human brain tissue from males and females aged 64-101 years old at the time of death that were cognitively normal or clinically diagnosed with Alzheimer's disease were used in this study. All individuals either had the APOE3/3 or APOE4/4 genotype (See Supplementary Table 1).
Recruitment	Subjects were not recruited specifically for this study. Samples are derived from the tissue repository of Banner Sun Health Research Institute.
Ethics oversight	Subjects included in this study were volunteers enrolled in AZSAND and its Brain and Body Donation Program ([BBDP]; www.brainandbodydonationprogram.org), at Banner Sun Health Research Institute (BSHRI) in metropolitan Phoenix, Arizona . All subjects signed informed consents, approved by BSHRI Institutional Review Boards, for both clinical assessment and autopsy for research purposes.

Note that full information on the approval of the study protocol must also be provided in the manuscript.

Field-specific reporting

Please select the one below that is the best fit for your research. If you are not sure, read the appropriate sections before making your selection.

☒ Life sciences ☐ Behavioural & social sciences ☐ Ecological, evolutionary & environmental sciences

For a reference copy of the document with all sections, see nature.com/documents/nr-reporting-summary-flat.pdf

Life sciences study design

All studies must disclose on these points even when the disclosure is negative.

Sample size	We did not use statistical methods to pre-determine sample sizes, but our sample sizes are similar to those reported in previous publications. Sample size was primarily determined by the availability of brain tissue.
Data exclusions	Single nucleus RNA-seq data that did not meet QC standards was excluded according to the following criteria: Count data was first screened for doublets with the Scrublet (0.2.3) Python package. Once each cell was doublet scored, we applied a separate doublet score threshold per sample to discard doublets from the data. Thresholds were identified between 0.15 and 0.5 per sample based on the sample-wise doublet score histograms (see Extended Figure 1a). We then applied the following standard filtering rules: we used the Scanpy (1.9.1) package to discard cells with (1) fewer than 500 genes or (2) less than total 1,000 reads or (3) more than 10% mitochondrial reads or (4) more than 10% ribosomal reads. Counts were then CPM scaled and log-normalized for downstream analysis.
Replication	Human brain nuclei were processed in independent batches, and results and quality control metrics were consistent across. Human staining data was performed on replicate individuals, with replicate numbers indicated in figure legends. Mouse data were repeated across 3 replicate animals and replication was succssful. Cell culture data was replicated in independent replicate wells, with replicate well numbers indicated in figure legends.
Randomization	For human brain nuclei, equal representation of control and AD samples were ensured in each batch of nuclei isolation. The exact samples from each disease group were then randomly selected for processing in each batch. Human subjects are matched for age, sex, and other covariates. Randomization not relevant for cell culture experiments.

Blinding	For experiments with human tissue, investigators were blinded to sample groups during nuclei isolation, RNA-sequencing library preparation and staining , imaging and image quantification. For experiments with mouse tissue investigators were blinded to sample groups during staining , imaging and image quantification. In general all analyses were performed by a blind observer wherever possible.

Reporting for specific materials, systems and methods

We require information from authors about some types of materials, experimental systems and methods used in many studies. Here, indicate whether each material, system or method listed is relevant to your study. If you are not sure if a list item applies to your research, read the appropriate section before selecting a response.

Materials & experimental systems

n/a	Involved in the study	
☐	☒	Antibodies
☐	☒	Eukaryotic cell lines
☒	☐	Palaeontology and archaeology
☐	☒	Animals and other organisms
☒	☐	Clinical data
☒	☐	Dual use research of concern
☒	☐	Plants

Methods

n/a	Involved in the study	
☒	☐	ChIP-seq
☐	☒	Flow cytometry
☒	☐	MRI-based neuroimaging

Antibodies

Antibodies used	Rabbit anti-Iba1 primary antibody (Wako, 019-19741)<br>Goat anti-Iba1 (Abcam, ab5076),<br>Rabbit anti-ACSL1 (Thermo Fisher, PA5-78713),<br>Mouse anti-β-amyloid (Cell Signaling Technologies, 15126)<br>Rabbit anti-PLIN2 (Proteintech, 15294-1-AP)<br>Chicken anti-MAP2 (ThermoFisher Scientific, PA1-10005),<br>Mouse anti-AT8 (ThermoFisher Scientific, MN1020)<br>Rabbit anti-Caspase-3 (Cell Signaling Technology, 9661)<br>Rabbit anti-LC3B Antibody (ThermoFisher Scientific, PA1-46286)<br>Alexa Fluor 488 (donkey anti-goat, Life Technologies Corporation, A-11055)<br>Alexa Fluor 555 (donkey anti-mouse, Life Technologies Corporation, A-31572)<br>Alexa Fluor 594 (donkey anti-rabbit, Life Technologies Corporation, A-21207)<br>Alexa Fluor 647 (donkey anti-chicken, Jackson Immunology, 703-605-155)
Validation	All antibodies were validated for the indicated applications by the manufacturer.

Eukaryotic cell lines

Policy information about cell lines and Sex and Gender in Research

Cell line source(s)	BV2 cells (E. Blasi), U-937 (ATCC). iPSC lines were a gift from Yadong Huang and details of the source of these iPSCs can be found in Wang, C. et al 2018.
Authentication	Cell line authentication was performed by the supplier, but not independently authenticated in our lab.
Mycoplasma contamination	Cell lines were tested for mycoplasma bi-annually and are negative.
Commonly misidentified lines (See ICLAC register)	No commonly misidentified lines were used.

Animals and other research organisms

Policy information about studies involving animals; ARRIVE guidelines recommended for reporting animal research, and Sex and Gender in Research

Laboratory animals	C57Bl/6 female mice, aged (19 months from NIA rodent colony), APOE3-KI/J20 female mice, APOE4-KI/J20 female mice (18-22 months). All mice housed in a 12h-12h light light-dark cycle at 68-73 degrees F, under 40-60% humidity.
Wild animals	This study did not involve wild animals.
Reporting on sex	Only female mice were used in this study.
Field-collected samples	This study did not involve field-collected samples.

Ethics oversight	All animal care and procedures complied with the Animal Welfare Act and were in accordance with institutional guidelines and approved by the institutional administrative panel of laboratory animal care at Stanford University.

Note that full information on the approval of the study protocol must also be provided in the manuscript.

Flow Cytometry

Plots

Confirm that:

☒ The axis labels state the marker and fluorochrome used (e.g. CD4-FITC).

☒ The axis scales are clearly visible. Include numbers along axes only for bottom left plot of group (a 'group' is an analysis of identical markers).

☒ All plots are contour plots with outliers or pseudocolor plots.

☒ A numerical value for number of cells or percentage (with statistics) is provided.

Methodology

Sample preparation	For staining with LipidSpot cells were passed through a 100 micron strainer and stained with LipidSpot-488(Biotium, 70065-T) and Cytox-Blue (Thermo) according to manufacturers instructions.
Instrument	Sony, MA900
Software	Sony MA900 Cell Sorter Software Version 3.1.1
Cell population abundance	For sorting for for ATAC-seq, RNA-seq, ctyokine assays, 1 million cells per replicate were sorted in to top 10% and bottom 10% Lipidspot intensity. For genome-wide CRISPR screens 220 million cells were sorted per replicate by top 10% and bottom 10% Lipidspot intensity. For iMG CRISPR screen 20 million were sorted per replicate by top 10% and bottom 10% Lipidspot intensity.
Gating strategy	Positive and negative gates were set using fluorescence minus one (FMO) background intensity controls. Fluorophores were chosen to minimize spectral overlap.

☒ Tick this box to confirm that a figure exemplifying the gating strategy is provided in the Supplementary Information.
